# Plant Hormone and Fatty Acid Screening of *Nicotiana tabacum* and *Lilium longiflorum* Stigma Exudates

**DOI:** 10.3390/biom13091313

**Published:** 2023-08-27

**Authors:** Maria Breygina, Dmitry Kochkin, Alexander Voronkov, Tatiana Ivanova, Ksenia Babushkina, Ekaterina Klimenko

**Affiliations:** 1 Department of Plant Physiology, Biological Faculty, Lomonosov Moscow State University, LeninskiyeGory 1-12, 119991 Moscow, Russia; 2 Russian Academy of Sciences, Timiryazev Institute of Plant Physiology, Botanicheskaya St. 35, 127276 Moscow, Russia

**Keywords:** *Nicotiana tabacum*, *Lilium longiflorum*, stigma exudate, plant reproduction, ABA, plant hormones, fatty acids, squalene

## Abstract

Pollen germination in vivo on wet stigmas is assisted by the receptive fluid—stigma exudate. Its exact composition is still unknown because only some components have been studied. For the first time, hormonal screening was carried out, and the fatty acid (FA) composition of lipid-rich (*Nicotiana tabacum*) and sugar-rich (*Lilium longiflorum*) exudates was studied. Screening of exudate for the presence of plant hormones using HPLC-MS revealed abscisic acid (ABA) in tobacco stigma exudate at the two stages of development, at pre-maturity and in mature stigmas awaiting pollination, increasing at the fertile stage. To assess physiological significance of ABA on stigma, we tested the effect of this hormone in vitro. ABA concentration found in the exudate strongly stimulated the germination of tobacco pollen, a lower concentration had a weaker effect, increasing the concentration did not increase the effect. GC-MS analysis showed that both types of exudate are characterized by a predominance of saturated FAs. The lipids of tobacco stigma exudate contain significantly more myristic, oleic, and linoleic acids, resulting in a higher unsaturation index relative to lily stigma exudate lipids. The latter, in turn, contain more 14-hexadecenoic and arachidic acids. Both exudates were found to contain significant amounts of squalene. The possible involvement of saturated FAs, ABA, and squalene in various exudate functions, as well as their potential relationship on the stigma, is discussed.

## 1. Introduction

In flowering plants, the stigma—the receptive, typically the terminal part of the pistil—receives pollen, ensuring its germination and directing pollen tubes (PTs) into the style [1]. Stigmas are divided into dry and wet [2], wet covered with a viscous liquid—exudate. Stigma exudate has a complex composition that can vary greatly in different plants, but it always includes proteins, carbohydrates, lipids, and low molecular weight substances [3]. Wet stigmas appear to fall into two principal types: stigmas with a lipophilic and hydrophobic surface exudate as the continuous phase, involving holocrine secretion and stigmas with a mucilaginous secretion of carbohydrates and proteins as the continuous phase, involving merocrine secretion mechanisms [4]. Martin [5] described in general terms the composition of stigma exudates of ten plant species from different taxonomic groups, finding that they all contained a lipid moiety.

In *Petunia hybrida*, the stigmatic exudate is primarily an oil free substance containing phospholipids, sterols, and free fatty acids (FAs) [6]; it has a high surface tension, as a single droplet residing on the stigma. Because of the high adhesiveness of stigma exudate, pollen easily sticks to it, and, presumably, a high content of hydrophobic molecules in the exudate protects pollen from being washed away by rain [6].

In classic studies on stigmas of *P. hybrida*, *Nicotiana tabacum,* and other plants with lipid-rich exudate, the technique of washing out stigma lipids with solvents was conventionally used [6,7,8,9,10,11]. The samples obtained were presented as the lipid fraction of stigma exudate; however, not only the exudate, but also the internalized lipids of stigma cells are washed out during the incubation of tissues in solvents. In *N. tabacum*, gas-chromatographic analysis has shown that such lipid component is formed of a large number of saturated and unsaturated FAs; major FAs were myristic (14:0), oleic (9–18:1), and an unidentified FA with a high number of C atoms [11]. The functions that the authors, as a discussion, assigned to FAs were (1) stigma protection from desiccation and (2) regulation of pollen hydration [12]. The latter has been confirmed in elegant experiments of Wolters-Arts et al. [13]. When *Petunia* exudate, rich in lipids, was applied to tobacco flowers with stigmas removed, PTs developed normally, but *Lilium* exudate, which contained mainly carbohydrates, could not support germination [13]. Moreover, pure triglycerides instead of exudate provided normal development of PTs [13].

A number of classical works analyzed carbohydrate composition of lily stigma exudate, so this subject has been studied in some detail [14,15,16], and the proteome of *Lilium longiflorum* and *Olea europaea* receptive fluid has been also reported [17]. A total of 51 and 57 proteins unique to these plants were identified in stigma exudate. The major group of exudate proteins includes catabolism enzymes: O-glycosylases, proteases, and lipases. The authors believe that these proteins cleave polymers of stigma exudate to oligomers and monomers, thereby facilitating their uptake by growing PTs. Among the low molecular weight exudate components, reactive oxygen species (ROS) are the most studied [18,19]. In tobacco stigmas, as the pistil matured, the level of both O^•^_2_^−^ and H_2_O_2_ in the exudate decreased markedly [20], whereas in lily, the level of total ROS increased [19].

Although there are already convincing data about ROS in the exudate, little is known about other physiological regulators—plant hormones—present on the wet stigma. However, their presence is quite probable, since pollen germination and membrane potential in plants with wet stigmas were reported to be sensitive to some hormones [21]. A stimulatory effect of abscisic acid (ABA) on H^+^-ATPase activity in petunia PT in vitro was mediated by an increase in [Ca^2+^]_cyt_ and ROS generation. The authors speculate that ethylene/ABA content of the stigma may control adhesion, hydration, and germination of pollen grains [22]. Indolylacetic acid (IAA), though sought, was not recovered from the stigmatic exudate of *Streptosolen jamesonii* (Solanaceae), which was studied using chromatography [23]. At the moment, there are data on the content of hormones in the stigma tissues, which are certainly easier to analyze. Thus, in *Vicia faba*, ABA content was highest in the ovary, but style + stigma also contained a remarkable amount of this hormone [24].Stigmas and anthers of tobacco contained more ABA than the other floral tissues [25], which matched the results of physiological tests. Extracts of these tissues had a strong inhibitory effect on seed germination. At the same time, ABA did not inhibit pollen germination and PT elongation in vitro [25]. Immunolocalization of IAA in tobacco stigmas showed that the highest signal was observed shortly after fertilization, and it was also high in juvenile stigmas. At the stage of fertility, the signal was weak [26]. In a study of *Paeonia* hybridization, it was found that during the whole fertilization process, IAA and gibberellic acid (GA_3_) contents of self-inbred pollinated pistils were significantly higher than those of hybrid incompatible pollinated pistils, whereas the ABA content in cross-pollinated pistils was higher than that of self-pollinated, indicating that high content of ABA was associated with hybrid incompatibility [27]. Measurement of endogenous hormone content in pistils of two cucumber lines showed a gradual increase during pollination, and three hormones (zeatin riboside, IAA and ABA) differed significantly between high-yield and low-strain lines [28].

After analyzing previously published data on the exudate biochemistry, we concluded that FA composition of exudate without lipids from stigma cells has been poorly studied before [23]. The hormonal composition of the exudate has also never been studied, apparently due to small volumes and low sensitivity of the equipment. Thus, the composition of FAs and hormones in stigma exudate has become the subject of our study, with tobacco and lily being suitable subjects with different types of exudate composition. The development of analytical techniques has allowed us to measure small amounts of substances collected from the stigma surface by non-invasive water wash.

## 2. Materials and Methods

### 2.1. Plant Material and Stigma Exudate Collection

Plants of *Nicotiana tabacum* L. var. Petit Havana SR1 were grown in a climatic chamber in controlled conditions (25 °C, 16 h light) in vermiculite. The plants were watered with salt solutions [29]. Cut branches of *Lilium longiflorum* L. var. White Heaven were purchased from a local shop.

Stigma maturity was assessed according to flower appearance and was divided into 4 stages(for details and appearance see [19]), where stage 1 was a juvenile stigma, stage 2 was a pre-mature stigma, stage 3 was a fully mature unpollinated stigma, and stage 4 was a pollinated stigma (the next day after pollination). Exudate was collected from stigmas of all stages by a “cap method”: A pipette tip containing 10 μL (tobacco) or an Eppendorf test tube containing 200 µL (lily) of distilled water was applied on the pistil and incubated for 30 min to wash the exudate off the stigma (25 °C). Then, the tip/tube containing the drop was carefully removed, and drops from different flowers of the same stage were placed in a cryo-tube and analyzed immediately (FAs) or frozen at −80 °C (hormones).

### 2.2. Chromato-Mass-Spectrometric Screening of Fatty Acids

FA methyl esters (FAMEs) were prepared according to a previously described method with slight modifications [30]. Margaric acid (17:0) (Sigma-Aldrich, Saint Louis, MO, USA, H3500) was added to exudate as an internal standard. The sample saponification was carried out in a boiling solution of 4% NaOH (Sigma-Aldrich, S5881) in methylalcohol/water (1:1, by volume). Then, the sample was evaporated to dryness using a rotary vacuum evaporator. H_2_O (1–2 mL) was added to the dried sample, and unsaponifiable FAs were washed out several times with hexane (Sigma-Aldrich, 439185) until clearness. Then, a few drops of methyl orange (Aronis, Geel, Belgium 9594) were added to the remaining water-soluble fraction, and it was acidified with 20% H_2_SO_4_ to a pink color. Then, FAs were extracted six times with hexane. The collected hexane was evaporated, and 3 mL of methanol (Sigma-Aldrich, 439193) and a few drops of acetyl chloride (Sigma-Aldrich, 00990) were added to the sample, and it was boiled for 1 h. Then, the sample was again evaporated, 1–2 mL of H_2_O and a few drops of methyl orange were added, and FAMEs were extracted six times with hexane. After that, the hexane was evaporated and 500 µL of benzene was added. The extract in benzene was pipetted onto a silicagel TLC plate, and a mixture of hexane/diethyl ether/glacial acetic acid (8:2:0.1, by volume) was used as a mobile phase. When the front moved to the top of the plate, the plate was removed and airdried for 1–2 min. Then, the plate was treated with a 0.001% solution of 2′,7′-dichlorofluorescein (Acros, Geel, Belgium 19153) in ethanol and airdried for 5–7 min. The FAME-containing zones were visualized in UV light (λ = 365 nm). Then, the sorbent from the FAME-containing zone of chromatographic plate was removed using a scalpel and transferred to a Schott glass filter, and the FAMEs were eluted from the sorbent by washing out with hexane six times. The FAMEs were analyzed via gas GC-MS on Agilent 7890A GC (Agilent, Santa Clara, CA, USA) with a quadrupole mass detector Agilent 5975C fitted with a 60 m capillary column DB-23 (inner diameter 0.25 mm, thickness of stationary phase, (50%-cyanopropyl)–methylpolysyloxane, 250 µm). The prepared FAMEs were separated under the following conditions: carrier gas, helium at 1 mL/min; sample volume, 1 µL; split ratio, 4:1 (in numerous analyses, splitless injection was used); and evaporator temperature, 260 °C. The oven temperature program was as follows: from 130 to 170 °Cat 6.5 °C/min, to 215 °C at 2.75 °C/min (25 min hold at this temperature), to 240 °C at40 °C/min (30 min hold at 240 °C). The operational temperature of the mass detector was set to 240 °C, and the ionization energy was set to 70 eV. To identify individual FAME species, NIST and Wiley search libraries and MSD ChemStation software, G1701EAE.0200.493 (Agilent, Santa Clara, CA, USA), were used, and the relative retention time and equal chain length (ECL) value were calculated for each peak [31]. Quantitative determination and identification of squalene were carried out from one sample with FAME using GC-MS method, as described [32].

### 2.3. Chromato-Mass-Spectrometric Screening of Phytohormones

The UPLC-ESI-MS method for the analysis of phytohormones and similar metabolites was developed. A representative UPLC-ESI-MS chromatogram of the model mixture of standard samples analyzed with the developed gradient elution system is presented in the Supplementary Figure S1. This technique allows for 15 min of gradient elution to carry out the simultaneous separation of different metabolites: polyamine derivatives (spermine), indole derivatives (IAA, indolebutyric acid (IBA), tryptophan and indigo derivatives), adenine derivatives, ABA, jasmonic acid, etc. Thus, this technique is convenient for screening the main phytohormones and structurally similar metabolites in extracts and other samples.

Sample preparation. Preparations of aqueous solutions of tobacco and lily pistil exudates were diluted with methanol 1:1 (by volume) before analysis and centrifuged at 15,294× *g* for 15 min.

Liquid chromatography-mass spectrometry, method 1(UPLC-ESI-MS, registration of positive ions). The analysis was performed on an ACQUITY UPLC H-Class PLUS chromatograph (Waters, Milford, MA, USA) equipped with a Xevo G2-XS TOF hybrid time-of-flight mass spectrometer (Waters, Milford, MA, USA). A sample in a volume of 0.1–1 µL was applied to a Titan C18 column (100 × 2.1 mm, 1.9 µm; Supelco, St. Louis, MO, USA). The column temperature (T) was 40 °C, and the volume flow rate of the mobile phase was 0.4 mL/min. A 0.1% (*v*/*v*) solution of formic acid in deionized water (solvent A) and a 0.1% (*v*/*v*) solution of formic acid in acetonitrile (solvent B) were used as the mobile phase. Chromatographic separation was carried out in gradient elution mode. During the analysis, the composition of the mobile phase changed as follows (B, % by volume): 0–1 min—5→15%, 1–5 min—15→30%, 5–11 min—30→38%, 11–15 min—38→65%, 15–15.5 min—65→95%. The analysis was carried out in the positive-ion detection mode (range *m*/*z* 100–1900). Ionization source parameters were as follows: ionization source T—150 °C, desolvation T—650 °C, capillary voltage (V)—3.0 kV, sample entry cone V—30 V, and nitrogen supply rate 1101 L/h. The obtained results were processed using the MassLynx 4.2 program (Waters, Milford, MA, USA).

The following standard samples were used to develop the LC-MS separation technique: Spermine, adenine, salicin,5-methyltryptophan (Serva, Heidelberg, Germany), hordenine, kinetin, IAA, jasmonic acid,indigo,2-phenylethyl-glucoside, picloram (4-amino-3,5,6-trichloropicolinic acid) (Sigma, Burlington, MA, USA), salicylic acid (Laverna, Moscow, Russia), trans-zeatin (FlukaChemie AG, Buchs, Switzerland, and Sigma, MA, USA), 6-benzylaminopurine, IBA, 2,4-dichlorophenoxyacetic acid (ICN Biomedicals Inc., Irvine, CA, USA), ABA (Sigma, MA, USA, and MP Biomedicals LLC, Irvine, CA, USA), and GA_3_ (Honeywell Riedel-de Haën AG, Seelze, Germany). Preparation of a model mixture of standards: A weighed sample of each standard (within 1–3 mg) was dissolved in 1 mL of a mixture of methanol–water (1:1, by volume); an aliquot (100 μL) of each standard stock solution was transferred into a volumetric flask, and the total volume of the solution was adjusted to 10 mL with methanol–water (1:1, *v*/*v*). The obtained solution of the standards model mixture was used for analysis. Relative standard deviation of the retention times of chromatographic peaks was ≤3%.

Liquid chromatography-mass spectrometry, method 2 (UPLC-ESI-MS, registration of positive and negative ions). HPLC-MS analysis was performed on a Waters ACQUITY UPLC chromatograph (Waters, Milford, MA, USA) equipped with a XEVO QTOF hybrid quadrupole time-of-flight mass spectrometer (Waters, Milford, MA, USA). The verification analysis was carried out in the positive- and negative-ion detection mode (range *m*/*z* 100–1200). Ionization source parameters were as follows: T—120 °C; desolvation T—250 °C; capillary V—3.0 kV; and sample input cone V—30 V.

Conditions for chromatographic separation were as follows: ACQUITY UPLC BEH Phenyl column (50 × 2.1 mm, 1.7 µm; Waters, Drinagh, County Wexford, Ireland), column T, 40 °C, and mobile phase flow rate, 0.4 mL/min. Mobile phase components were as follows: 0.1% (*v*/*v*) formic acid in water (solvent A) and 0.1% (*v*/*v*) formic acid in acetonitrile (solvent B). All analyzes were performed using a gradient elution mode. The composition of the mobile phase changed as follows (solvent B, % by volume): 0–1 min—15%, 1–5 min—15→30%, 5–15 min—30→38%, 15–15.5 min –38→45%, 15.5–23 min—45%, 23–23.5 min—45→95%.

Quantitative analysis of ABA in aqueous solutions of tobacco exudate was performed by the method of external calibration with ABA standard sample (Sigma, MA, USA). In the working range of concentrations (48–0.048 µg/mL), the calibration curve was approximated by a straight line with R^2^ above 0.99999. The relative standard deviation of retention times and areas of chromatographic peaks of ABA did not exceed 3 and 5%, respectively. The lowest detectable concentration of ABA is 5 ng/mL. The results were processed using the MassLynx 4.2 software (Waters, Milford, MA, USA).

### 2.4. Pollen Collection and Germination In Vitro

For pollen collection, the anthers were removed from the flowers on the eve of opening (stage 2) and dried in a thermostat for 2 days, after which the pollen was collected with a specially equipped vacuum cleaner. Dry pollen was stored at −20°C. Pollen germination efficiency was assessed after 1 h of cultivation at 25 °C in standard medium containing 0.3 M sucrose, 1.6 mM H_3_BO_3_, 3 mM Ca(NO_3_)_2_, 0.8 mM MgSO_4_, and 1 mM KNO_3_ in 25 mM MES-Tris buffer, pH 5.8 at 2 mg pollen/mL. Before cultivation, pollen was pre-hydrated in a humid atmosphere for 2 h. Germinated pollen was fixed with 2% paraformaldehyde in 50 mM Na-phosphate buffer, pH 7.4 for minimum 30 min at 4 °C. Between 500 and 900 pollen grains from each suspension were examined using microscopy (120×) for germination. ABA was added at the beginning of incubation from stock solution (1 mM). To prepare the stock solution, dry hormone was dissolved in a small volume of 70% alcohol, after which it was adjusted with water to the required volume. The final concentrations were 0.02, 0.2, and 2 µM. Squalene was added to pollen suspensions from stock solution in hexane (10% *m*/*v*) or diluted in water. The final concentrations were 1 µg/mL–100 µg/mL.

### 2.5. Data and Statistical Analyses

All experiments were performed in triplicate with at least three independent executions. The data are provided as the means ± SEM. Statistical analysis for FAs was performed using one-way ANOVA followed by post hoc analysis using Tukey’s honest significant difference (HSD) for unequal N tests. (*—*p* < 0.05, **—*p* < 0.01) (STATISTICA 10, StatSoft, Tulsa, OK, USA). To characterize the saturation level of lipid FAs, the unsaturation index (UI) was calculated [33].

For pollen germination, significant difference was determined using Origin Lab software 9.7 (Northampton, MS, USA) according to Mann–Whitney test (*—*p* < 0.05, **—*p* < 0.01).

## 3. Results

Tobacco and lily are plants with wet stigmas. Visible exudate production in lily starts earlier, i.e., the stigmas are already moist at stage 1. In tobacco, visible moisture appears at stage 2; however, earlier studies show that the wash from the stigma at stage 1 contains active components [20]. Therefore, we considered all stages from both subjects for hormonal screening. To analyze the FA composition, we collected exudate from stages 2 and 3, since visually the volume of exudate was stable and the maximum among the stages.

### 3.1. Fatty Acids of Tobacco Stigma Exudate

The total lipids of tobacco stigma exudate were represented by 11 types of individual C_14–24_FAs. The main ones were palmitic (16:0), stearic (18:0), 9–18:1, and linoleic (9,12–18:2) acids (Table 1). These four FAs accounted for more than 80% of the total FAs. The relative content of minor FAs: 14:0, pentadecylic (15:0), palmitoleic (9–16:1), arachidic (20:0), behenic (22:0), and lignoceric (24:0), ranged from 1.04 to 3.03%. Only 14-hexadecenoic (14–16:1) acid was present at less than 1%.

Very-long-chain FAs (VLCFA) of total lipids of tobacco stigma exudate accounted for ≈4.47% of the total amount of FAs and were represented by three individual types of FAs. Almost half of the total VLCFAs was 24:0.

The unsaturation index (UI) of tobacco stigma exudate lipids was 0.568. Such a low value can be explained by the high proportion of saturated (almost 50%) and monoene FAs (≈44%), a small amount of dienes (≈6.5%), and absence of other types of polyene FAs.

### 3.2. Fatty Acids of Lily Stigma Exudate

The total lipids of lily stigma exudate were represented by 17 types of individual C_14–26_FAs. The main ones were 16:0, 7-hexadecenoic (7–16:1), 18:0, and 9–18:1 acids (Table 1). These four FAs accounted for more than 70% of the total FAs. Relative content of minor FAs: 14:0, 15:0, 14–16:1, cis-10-heptadecenoic (10–17:1), 9,12–18:2, 20:0, gadoleic (11–20:1), 22:0, 24:0, cerotic (26:0), ranged from 1.15 to 3.63% for the above FAs. Vaccenic (11–18:1), α-linolenic (9,12,15–18:3), and pentacosylic (25:0) acids were present as less than 1%. VLCFAs of total lipids accounted for ≈12.54% of the total amount and were represented by six individual types. Half of them were 11–20:1 and 24:0 acids.

The UI of lily stigma exudate lipids was 0.453. Such a low value is due to a large proportion of saturated (more than 50%) and monoene (about 37%) FAs and a small amount of polyene (≈4%) FAs.

Comparing the composition of FAs in lily and tobacco stigma exudate, one can conclude that tobacco exudate contained significantly more 14:0, 9–18:1, and 9,12–18:2, which is also expressed in a significantly higher UI relative to lily exudate lipid values. Lily stigma exudate lipids contained significantly more 14–16:1 as well as 20:0, which belongs to VLCFA.

In addition to FAs, squalene was also found in the analyzed samples, and it was present in both tobacco and lily stigma exudates (Table 1). The squalene content of the exudate can be divided by the average weight of the stigma, which was determined in the experiment: for tobacco, it averages to 2.7 mg, for lily to about 60 mg, that is, per 1 mg of stigma mass, the content of squalene is 0.18 ng for tobacco, and 0.29 ng for lily.

### 3.3. Hormones of Stigma Exudate

Original ultra-performance liquid chromatography-electrospray ionization-tandem mass spectrometry (UPLC-ESI-MS) method was developed to analyze main plant hormones and metabolites with similar structure. The proposed method (method 1) allows, in 15 min of gradient elution, the simultaneous separation of a wide range of metabolites: polyamine derivatives (spermine), indole derivatives (IAA, IBA, tryptophan, and indigo derivatives), adenine derivatives, ABA, jasmonate, etc. (Figure S1). Thus, this technique can be used for screening of plant hormones in extracts and other liquids. We used this method to identify hormones in aqueous solutions of stigma exudates of tobacco and lily at four different stages (1–4) of stigma development.

**Figure 1:**
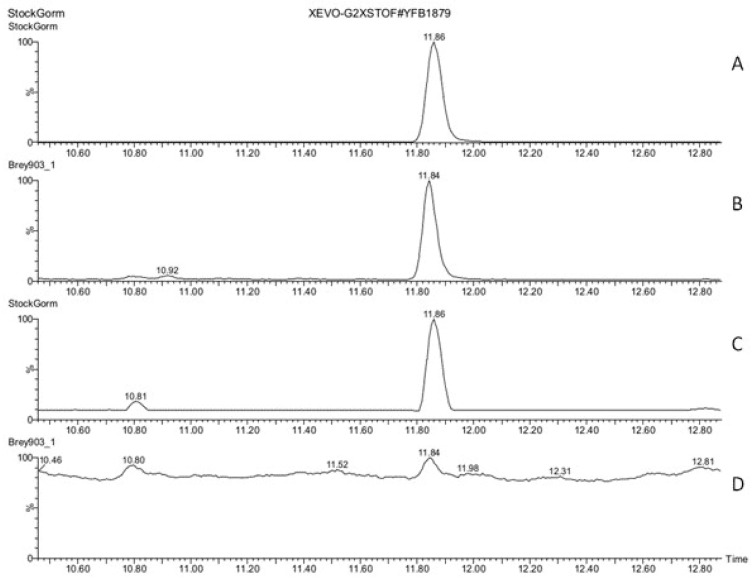
ABA in the stigma exudate of tobacco (stage 3). UPLC-ESI-MS chromatograms (total ion current, positive-ion mode) obtained by method 1 of a model mixture of standard samples of phytohormones and related metabolites (**A**,**C**) and tobacco stigma exudate (**B**,**D**). (**A**,**B**)—Results of signal filtering by *m*/*z* value (value 287.3) of [M + Na]^+^ ion of ABA; (**C**,**D**)—primary signal. X—t, min; Y—detector signal, relative intensity (RI), %.

**Figure 2:**
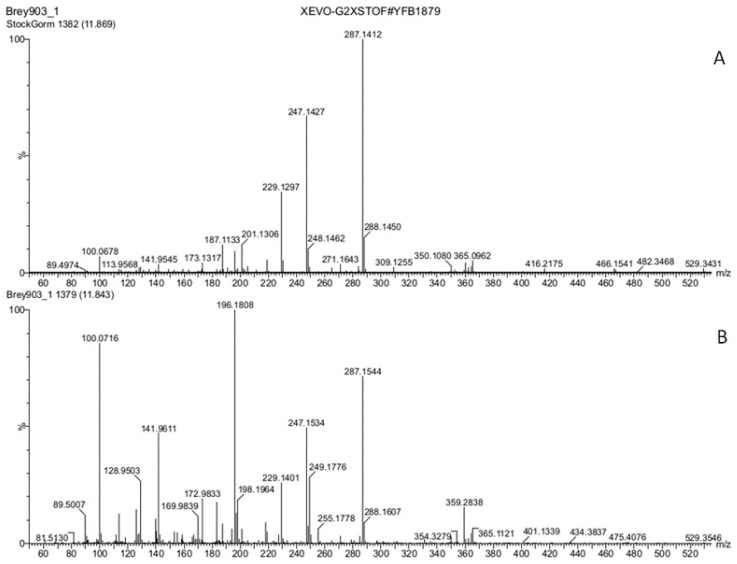
Mass spectra (positive ions; method 1) of the chromatographic peak of ABA standard sample (**A**) and the chromatographic peak with a retention time of 11.84 min in the chromatogram of tobacco stigma exudate (stage 3) (**B**). X—*m*/*z*; Y—detector signal, RI, %.

The results of screening of tobacco stigma exudate (stage 3) for auxins (IAA, IBA), cytokinins (6-benzylaminopurine (BAP), trans-zeatin), and jasmonic acid (Figures S2–S7) showed that these phytohormones were absent in detectable amounts in the studied samples, as well as GA_3_, salicylate, and spermine. The exception was ABA, which was identified in tobacco stigma exudate (stages 2 and 3) based on the similarity with the standard sample in chromatographic and mass spectrometric characteristics (Figure 1, Figure 2 and Figure S8).

To verify the correctness of the performed identification, a mixture of standards and aqueous solution of tobacco stigma exudate were analyzed on another device under different chromatographic conditions (method 2). The obtained results (Figures S9 and S10) confirm the presence of ABA in tobacco stigma exudate. The structural identity of the discovered metabolite with ABA was also confirmed by comparing the experimental and calculated exact monoisotopic *m*/*z* values for the most intense ions in the corresponding mass spectra: for the adduct ion [M + Na]^+^, the experimental value *m*/*z* was 287.1264 (calculated value for C_15_H_20_O_4_Na—287.1259); and for deprotonated [M-H]^−^ molecule, the experimental value was *m*/*z* 263.1263 (calculated value for C_15_H_19_O_4_—263.1283).

**Figure 3:**
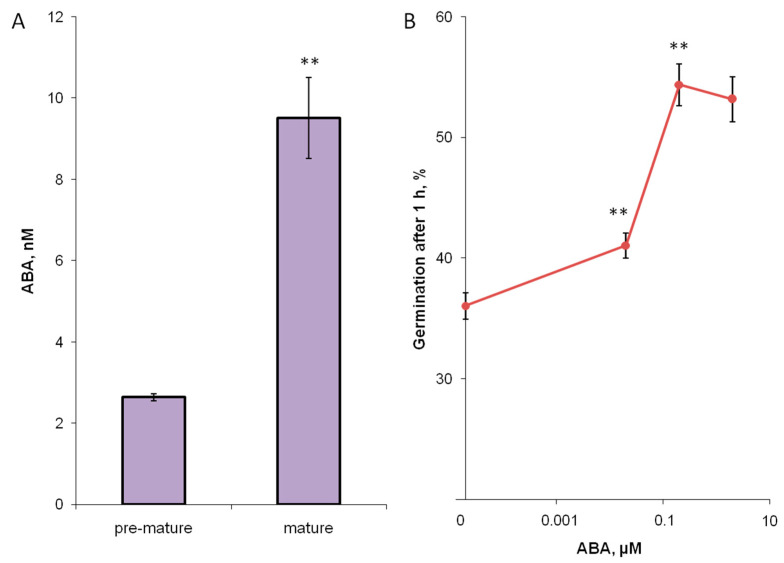
ABA in tobacco stigma exudate washout (**A**) and its effect on pollen germination in vitro (**B**). The level of ABA significantly differs between the two developmental stages (pre-mature = 2, mature = 3, see Methods). At stages 1 (juvenile) and 4 (pollinated), ABA was absent in detectable amounts. ABA added to pollen (collected from stage 3) suspensions germinating in vitro significantly stimulated germination in concentrations close to those found in the collected stigma exudate; **—*p*< 0.01 (Mann–Whitney test).

Next, a quantitative analysis of ABA in stigma exudates was carried out (Figure 3A). At stage 3, the content of ABA was significantly higher than at stage 2. At the stages 1 and 4, ABA was absent in the exudate collected from the same plants.

To assess the physiological significance of ABA in the tobacco stigma exudate, we tested the effect of this hormone in vitro. We calculated that if in the washout of the exudate from the stigma that we analyzed with UPLC-MS, ABA concentration was about 10 nM at the stage of fertility (Figure 3A), and in the pure exudate, it is approximately 20 times higher, i.e., 200 nM. We considered this concentration for in vitro testing, as well as an order of magnitude lower and higher. The concentration closest to that in the exudate strongly stimulated germination of tobacco pollen, a lower concentration had a weaker effect, although the stimulation was also significant (Figure 3B). Increasing the concentration by an order of magnitude did not lead to an additional enhancement of the effect.

Phytohormones (including ABA) were absent in detectable amounts in aqueous solutions of lily pistil exudates (Figure S11).

**Figure 4:**
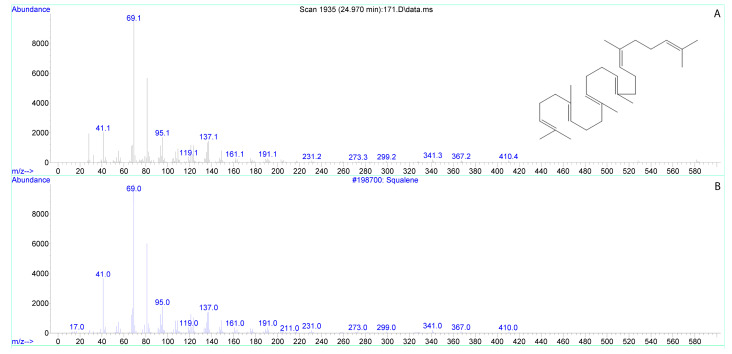
Mass spectra of squalene in the sample of lily stigma exudate (**A**) and in NIST search libraries (**B**).

We tested the possible stimulation of germination in vitro with squalene (Table 1, Figure 4) over a wide range of concentrations, only weak stimulation (5%) was found with concentration 100 times higher than in tobacco exudate, and lower concentrations were ineffective. Since squalene is insoluble in water, it was added both in pure form and from a stock solution, adding the solvent (hexane) into control suspension (Figure S12).

## 4. Discussion

For the first time, we analyzed the FA composition of stigma exudate of *N. tabacum* and *L. longiflorum* without admixture of stigma cells. The FA composition of lily exudate, as well as its stigma, has not been previously studied, since it belongs to the “carbohydrate type”, that is, it contains mainly oligo- and monosaccharides [15,16,34]; for tobacco and other species with lipid-rich exudate, the total FA composition of the stigma has been analyzed [6,7,8,9,10,11]. Here, we report that the content of saturated FAs in both stigma exudates was very high, which strikingly distinguished it from the typical composition of plant membrane lipids, which are characterized by the predominance of unsaturated FAs [35,36]. So, for tobacco exudate, the UI was 0.568, and for lily, it was 0.453, that is, even lower than in the pollen coat of the same species, which was about 0.7, due to the high content of 16:0,22:0,and other saturated FAs [37]. Such low UIs show a large proportion of saturated (more than 50%) and monoenoic FAs (37–44%) and a small amount of polyenoic FAs in the lipids of both stigma exudates. This interesting pattern has been revealed by us for the first time and may indicate the special functions of exudate lipids associated with a high degree of saturation.

In the literature, the following functions are attributed to stigma exudate: regulation of pollen germination [13,38], protection of the stigma from desiccation [11,39], adhesion of pollen and protection from washing off [6,40], and attraction of pollinators and their nutrition [41,42,43,44]. The need for natural exudate lipids or artificial lipids of similar composition for pollen hydration and germination has been shown in vivo [13,45] and in vitro [46].The composition of artificial lipids applied to tobacco pistil strongly influenced the rate of pollen hydration. The fastest hydration was observed in the case of tricaprylin, that is, triglyceride with saturated FAs [38], which is in good agreement with our data.

We also screened plant hormones in stigma exudate and found a number of important features. We cannot discuss this result in comparison to other exudates because there are no data on hormones in these fluids (with the exception of the work of Shuel, who looked for IAA and confirmed its absence [23]). However, it makes sense to compare the hormonal composition of the exudate with other floral secretions such as nectar. Here, ABA was detected in tobacco, but not in lily stigma exudate. Previously, ABA was found in the nectar of three species of dicotyledonous plants: *Brassica napus*, *Lamium album* [47], and *Viciafaba* [48]. Nectars of monocots, for example, *Cymbidium* sp. or *Sanseveria trifasciata*, did not contain ABA [47]. These observations confirm the relationship between stigma exudate and nectar, which was previously traced through the movement of labeled carbon [23], and also indicate the possible features of the representatives of the two taxa associated with the hormonal regulation of reproduction.

ABA found in the stigma exudate could play a significant role in controlling pollen germination. Since we could not obtain stigma exudate without ABA content, and we considered the complete removal of exudate or stigma as a dubious experiment, we tested the possible effect of this hormone on pollen of the same species in vitro, and the concentration found on the stigma turned out to be very effective in stimulating germination. These data are consistent with the accumulation of this hormone during stigma maturation (stage 2 < stage 3), and its absence in detectable amounts on juvenile stigmas and after pollination, when pollen germination is finished. These data demonstrate the role of ABA in germination control in vivo and are also consistent with previously found effects for other species: ABA, as well as some concentrations of IAA and GAs, activated petunia pollen germination and cytokinin and ethylene inhibited it [49].Other hormones are absent in the exudate, but they can be synthesized in the pistil tissues and participate in the regulation of PT growth at later stages compared to ABA. So, it has been reported that IAA is present in the pistil tissues and acts as a guiding factor for PT growth in the style [26,50]. 

The fact that ABA is the first hormone encountered by pollen upon landing on tobacco stigma exudate is thought to be related to its role in water redistribution in plant cells and tissues. This hypothesis is consistent with the content of endogenous hormones in tobacco and petunia pollen: the maximum of ABA was detected before pollen activation; later, the content of this hormone decreased [51,52], and the intracellular localization also changed [51].

It is unclear whether there is a relationship between the accumulation of ABA and FAs in stigma exudate, but such a relationship has been shown for a number of objects, including seeds, which naturally accumulate this hormone; additional ABA treatment promoted the expression of FAD2 and other genes involved in FA biosynthesis, which resulted in the accumulation of linoleic acid in oil palm mesocarp [53] and upregulated expression of ABI3, SAD6, FAD2, and KCS1-like genes and enhanced 9–18:1 and 9,12–18:2 accumulation in developing Siberian apricot seeds [54]. Tobacco exudate containing ABA also contained almost twice as much 9–18:1 and 9,12–18:2 as compared to lily exudate, in which ABA was absent, but so far this can only be speculated on.

The mechanical functions of the exudate, associated with the adhesion of pollen and protection from drying [11,39], as well as the preservation of its teardrop shape on the stigma, most likely determine the FA composition of exudate lipids. It can be assumed that saturated FAs provide a high surface tension [55] and better protect the liquid from evaporation, allowing it to perform its regulatory functions discussed above. The mechanical function of lipids has been discussed for nectar, the evaporation of which is reduced due to the presence of lipids on the surface of the drop [56]. In this, one can trace the similarity with the stigma exudate. A possible reason for the high proportion of saturated FAs in the exudate is the low activity of desaturases, which is due to the fact that these lipids are not embedded in membranes and do not accumulate inside cells, but are produced, like wax, on the cell surface. Waxes also contain predominantly saturated FAs, but with a long chain [57].

In the exudates of both species, in addition to lipids and hormones, squalene was present in appreciable amounts. Data on the presence of this substance on stigmas have been obtained for the first time. Squalene is a precursor in sterol biosynthesis in plant cells and participates in their response to ABA [58].A direct link between squalene and ABA is less likely, since squalene, unlike the hormone, is found in both species. However, such a connection cannot be ruled out. There are cases reported when an increase in endogenous formation of squalene [59] or inhibition of enzymes metabolizing squalene [60] led to a change in the expression of enzymes of ABA biosynthesis and contributed to an increase in the content of this phytohormone in vegetative plant tissues. In turn, ABA-responsive elements (ABREs) are present in squalene synthase genes [61], genes for enzymes that metabolize squalene [62,63], and genes for transcription factors of the basic helix-loop-helix (bHLH) family, which regulate the synthesis of squalene and triterpenoids [64]. However, the effect of ABA on squalene biosynthesis is complex and ambiguous. Examples are known wherein exogenous ABA both enhances [65,66] and suppresses [67,68] the accumulation of squalene in vegetative plant tissues.

In the tobacco stigma, the functions of ABA and squalene on the stigma appear to be different, as ABA stimulates pollen germination while squalene does not. It is more likely that squalene is a precursor of other substances and provides protective, mechanical properties or controls ROS balance of the exudate. Squalene epoxidase SQE1 was found to be a mediator that controls ROS production during ABA-dependent stomatal closure and root hair growth in *Arabidopsis* [58]. Since, as previously shown for tobacco, ROS balance in stigma exudate is important for pollen germination in vivo [20], there may be a relationship between these components of the receptive fluid. Squalene has antioxidant properties, in particular, the ability to quench free radicals [69], and thus can participate in maintaining ROS balance during pollination.

## 5. Conclusions

For the first time, FA and hormonal screening of *Nicotiana* and *Lilium* stigma exudate was carried out. HPLC-MS revealed ABA in tobacco stigma exudate at pre-maturity and in mature stigmas, increasing at the fertile stage. Testing the physiological significance of ABA in vitro showed that its main function may be the regulation of pollen germination on the pistil surface. GC-MS showed that both in tobacco and lily lipids of the stigma exudate contain a large percent of saturated FAs. The supposed function of these FAs is a high surface tension, which would protect the exudate droplet from being washed away, evaporated, or shaken off. Both exudates were found to contain significant amounts of squalene. As squalene does not significantly stimulate tobacco pollen germination in vitro, we hypothesize that it has a number of other functions, including protective, synthetic, mechanical, or associated with ROS balance on stigma.

## Figures and Tables

**Table 1:** Fatty acid composition of tobacco and lily stigma exudate from stages 2 and 3 (mass % of the amount of FAMEs). Statistical analysis was performed using one-way ANOVA followed by post hoc analysis using Tukey’s honest significant difference for unequal *N* tests (*—*p* < 0.05, **—*p* < 0.01).

Fatty Acid	Exudate	
	Lily (*Lilium longiflorum* L.)	Tobacco (*Nicotiana tabacum* L.)
14:0	1.90 ± 0.07	3.03 ± 0.23 *
15:0	3.63 ± 0.26	2.79 ± 0.56
16:0	27.36 ± 3.43	24.08 ± 1.80
7–16:1	8.42 ± 0.69	– (not detected)
9–16:1	–	4.44 ± 0.44
14–16:1	1.78 ± 0.07	0.93 ± 0.02 *
10–17:1	1.78 ± 0.07	–
18:0	16.86 ± 0.68	15.33 ± 1.97
9–18:1	21.34 ± 0.97	38.47 ± 1.59 **
11–18:1	0.56 ± 0.13	–
9,12–18:2	3.01 ± 1.71	6.47 ± 0.56 *
9,12,15–18:3	0.81 ± 0.55	–
20:0	2.69 ± 0.47	1.04 ± 0.10 *
11–20:1	3.01 ± 0.50	–
22:0	1.88 ± 0.93	1.35 ± 0.83
24:0	3.22 ± 1.69	2.08 ± 0.02
25:0	0.58 ± 0.68	–
26:0	1.15 ± 0.47	–
UI	0.453 ± 0.010	0.568 ± 0.030 *
∑VLCFAs, %	12.53 ± 3.25	4.47 ± 1.91 *
ngsqualeneon 1 stigma	17.66 ± 1.19	0.51 ± 0.03 *

## Data Availability

Original data are contained within this article and supplementary material.

## Supplementary Materials

The following supporting information can be downloaded at: https://www.mdpi.com/article/10.3390/biom13091313/s1, Figure S1: A representative UPLC-ESI-MS chromatogram (total ion current, base peak ion (BPI) mode, positive ions; method 1) of a model mixture of standard samples of phytohormones and structurally similar metabolites. Identification of chromatographic peaks by retention time: spermine (retention time—0.42 min), adenine (0.76 min), hordenine (1.65 min), salicylic acid (3.40 min), trans-zeatin (3.76 min), kinetin (5.28 min), 2,4-dichlorophenoxyacetic acid (5.94 min),picloram (6.38 min), 2-phenylethyl-glucoside (7.34 min), 6-benzylaminopurine (7.44 min), indole-3-acetic acid (9.48 min), salicin (10.81 min), ABA (11.86 min), indole-3-butyric acid (13.01 min), jasmonic acid (13.26 min), 5-methyltryptophan (13.56 min), and indigo (14.37 min). X—t, min; Y– detector signal, relative intensity (RI), %. Figures S2–S6: Screening for indole-3-acetic acid (S2), indole-3-butyric acid (S3), 6-benzylaminopurine (S4), trans-zeatin (S5), and jasmonic acid (S6), in tobacco stigma exudate (stage 3). UPLC-ESI-MS chromatograms (total ion current (TIC) and BPI modes, positive ions; method 1) of tobacco stigma exudate (lower panel) and a model mixture of standard phytohormone samples (second panel from the bottom), as well as the results of filtering these signals to the *m*/*z* value of the [M + H]^+^ ion of the hormone (the third and fourth panels from the bottom for the exudate and the model mixture of standards, respectively). X—t, min; Y—detector signal, RI, %; Figure S7: Screening for the presence of major phytohormones in tobacco stigma exudate (stage 3, sample collected in the summer of 2022). UPLC-ESI-MS chromatogram (total ion current (TIC and BPI modes), positive ions; method 1) of exudate (first and second panel from the bottom), results of filtering the TIC signal by *m*/*z* value for the following ions: m/ z 220.1—[M + H]^+^ trans-zeatin ion (third panel from the bottom); *m*/*z* 176.1—[M + H]^+^ion of indole-3-acetic acid (panel fourth from bottom); *m*/*z* 226.2—ion [M + H]^+^ 6-benzylaminopurine (fifth panel from the bottom); *m*/*z* 204.2—[M + H]^+^ ion of indole-3-butyric acid (sixth panel from the bottom); *m*/*z* 287.1—[M + Na]^+^ ion of ABA (panel seventh from the bottom). X—t, min; Y—detector signal, RI, %. The chromatographic peak of ABA has a retention time of 11.82 min; Figure S8: ABA in the stigma exudate of tobacco (stage 2). UPLC-ESI-MS chromatogram (total ion current, positive-ion mode) obtained by Method 1 of tobacco stigma exudate. Upper panel—results of signal filtering by *m*/*z* value (value 287.1) of [M + Na]^+^ ion of ABA; Lower panel—primary signal. X—t, min; Y—detector signal, relative intensity (RI), %; Figure S9: Screening for ABA in tobacco stigma exudate (method 2). The UPLC-ESI-MS chromatograms (total ion current, negative ions) of tobacco stigma exudate (second panel from the bottom) and the model mixture of standard phytohormone samples (bottom panel) are presented, as well as the results of filtering these signals by *m*/*z* value (value 263.1) [M-H]^−^ ABA ion (third and fourth panels from the bottom for the model mixture of standards and exudate, respectively). X—t, min; Y—detector signal, RI, %; Figure S10: Mass spectra of the chromatographic peak of the standard sample of ABA (lower panel) and the chromatographic peak with a retention time of 3.49 min in the chromatogram of tobacco stigma exudate (upper panel). Method 2. X—*m*/*z*; Y—detector signal, RI, %; Figure S11: Screening for the presence of major phytohormones in lily stigma exudate (stage 3). UPLC-ESI-MS chromatogram (TIC mode, positive ions; method 1) of the exudate (first panel from the bottom), results of filtering the TIC signal by *m*/*z* value for the following ions: *m*/*z* 220.1—ion [M + H]^+^ trans-zeatin (panel second from bottom); *m*/*z* 176.1—[M + H]^+^ ion of indole-3-acetic acid (third panel from the bottom); *m*/*z* 226.2—ion [M + H]^+^ 6-benzylaminopurine (fourth panel from the bottom); *m*/*z* 204.2—[M + H]^+^ ion of indole-3-butyric acid (panel fifth from the bottom); and *m*/*z* 287.1—[M + Na]^+^ ion of ABA (sixth panel from the bottom). X—t, min; Y—detector signal, RI, %; Figure S12: Squalene effect on tobacco pollen germination in vitro. Squalene added to pollen suspensions germinating in vitro in concentration close to the one found in stigma exudate (1 µg/mL had no effect on germination efficiency, and 100 µg/mL had a weak stimulating effect both with pure squalene in water and squalene diluted in hexane). *—*p*< 0.05 (Mann–Whitney test).

## References

[1] Shivanna K.R. (2020). The Pistil: Structure in Relation to Its Function. Reproductive Ecology of Flowering Plants: Patterns and Processes.

[2] Heslop-Harrison Y., Shivanna K.R. (1977). The receptive surface of the angiosperm stigma. Ann. Bot..

[3] Rejón J.D., Delalande F., Schaeffer-Reiss C., Carapito C., Zienkiewicz K., Alché J.d.D., Rodríguez-García M.I., Van Dorsselaer A., Castro A.J. (2014). The plant stigma exudate: A biochemically active extracellular environment for pollen germination?. Plant Signal. Behav..

[4] Knox R.B. (1984). Pollen-pistil interactions. Cellular Interactions.

[5] Martin F.W. (1969). Compounds from the stigmas of ten species. Am. J. Bot..

[6] Konar R.N., Linskens H.F. (1966). Physiology and biochemistry of the stigmatic fluid of Petunia hybrida. Planta.

[7] Tiezzi A., Focardi S., Ciampolini F., Oresti M. (1981). La componentelipidicadell’essudatostigmatico di citrus limon (L.) burm. G. Bot. Ital..

[8] Dumas C. (1977). Lipochemistry of the progamic stage of a self-incompatible species: Neutral lipids and fatty acids of the secretory stigma during its glandular activity, and of the solid style, the ovary and the anther in *Forsythia intermedia* Zab. (Heterostylic species). Planta.

[9] Knox R.B., Kenrick J., Jobson S., Dumas C. (1989). Reproductive Function in the Mimosoid Legume *Acacia retinodes*: Ultrastructural and Cytochemical Characteristics of Stigma Receptivity. Aust. J. Bot..

[10] Hawker J.S., Sedgley M., Loveys B.R. (1983). Composition of Stigmatic Exudate, Nectar and Pistil of Watermelon, *Citrulluslanatus* (Thunb.) Matsum. &Nakai, Before and After Pollination. Funct. Plant Biol..

[11] Cresti M., Keijzer C.J., Tiezzi A., Ciampolini F., Focardi S. (1986). Stigma of Nicotiana: Ultrastructural and biochemical studies. Am. J. Bot..

[12] Vasil I.K. Histology and physiology of pollen germination and pollen tube growth on the stigma and in the style. Proceedings of the International Symposium on Fertilization in Higher Plants.

[13] Wolters-Arts M., Lush W.M., Mariani C. (1998). Lipids are required for directional pollen-tube growth. Nature.

[14] Labarca C., Kroh M., Loewus F. (1970). The composition of stigmatic exudate from *Lilium longiflorum*. Plant Physiol..

[15] Labarca C., Loewus F. (1972). The Nutritional Role of Pistil Exudate in Pollen Tube Wall Formation in *Lilium longiflorum*: I. Utilization of Injected Stigmatic Exudate 1. Plant Physiol..

[16] Miki-Hirosige H., Hoek I.H.S., Nakamura S. (1987). Secretions from the pistil of *Lilium longiflorum*. Am. J. Bot..

[17] Rejón J.D., Delalande F., Schaeffer-Reiss C., Carapito C., Zienkiewicz K., de Dios Alché J., Rodríguez-García M.I., Van Dorsselaer A., Castro A.J. (2013). Proteomics profiling reveals novel proteins and functions of the plant stigma exudate. J. Exp. Bot..

[18] Breygina M., Klimenko E. (2020). ROS and ions in cell signaling during sexual plant reproduction. Int. J. Mol. Sci..

[19] Breygina M., Luneva O., Schekaleva O., Lazareva N., Babushkina K., Kirilyuk I.A. (2023). Pattern of ROS generation and interconversion on wet stigmas in basal and divergent angiosperms. Plant Growth Regul..

[20] Breygina M., Schekaleva O., Klimenko E., Luneva O. (2022). The balance between different ROS on tobacco stigma during flowering and its role in pollen germination. Plants.

[21] Kovaleva L.V., Zakharova E.V., Minkina Y.V., Timofeeva G.V., Andreev I.M. (2005). Germination and In Vitro growth of petunia male gametophyte are affected by exogenous hormones and involve the changes in the endogenous hormone level. Russ. J. Plant Physiol..

[22] Zakharova E.V., Khaliluev M.R., Kovaleva L.V. (2022). Hormonal Signaling in the Progamic Phase of Fertilization in Plants. Horticulturae.

[23] Shuel R.W. (1961). Influence of reproductive organs on secretion of sugars in flowers of *Streptosolenjamesonii*, Miers. Plant Physiol..

[24] Dathe W., Sembdner G. (1981). Endogenous Plant Hormones of the Broad Bean, Viciafaba L. III. Distribution of Abscisic Acid and Gibberellins in the Pistil at Anthesis. Biochem. Physiol. Pflanz..

[25] Matsuzaki T., Koiwai A. (1986). Germination inhibition in stigma extracts of tobacco and identification of MeABA, ABA, and ABA-γ-d-Glucopyranoside. Agric. Biol. Chem..

[26] Chen D., Zhao J. (2008). Free IAA in stigmas and styles during pollen germination and pollen tube growth of Nicotiana tabacum. Physiol. Plant..

[27] Yang M., Yarra R., Zhang R., Zhou L., Jin L., Martin J.J.J., Cao H. (2022). Transcriptome analysis of oil palm pistil during pollination and fertilization to unravel the role of phytohormone biosynthesis and signaling genes. Funct. Integr. Genomics.

[28] Ren M.J., Zhou X.Y. (2017). Change of cucumber seed yield lines endogenous hormone during the process of double fertilization. J. Northeast Agric. Univ..

[29] Nitsch J.P. (1965). Deux espacesphotoperiodiques de jours courts: *Plumbago indica* L. et *P. zeyelanica* L.. Bull. Bot. Fr..

[30] Ivanova T.V., Voronkov A.S., Kumakhova T.K., Tsydendambaev V.D. (2020). Distinguishing Features of Fatty Acid Content and Composition in Total Lipids of Malus orientalisUglitzk. Pericarp. Russ. J. Plant Physiol..

[31] Voronkov A.S., Ivanova T.V., Kumachova T.K. (2022). The features of the fatty acid composition of Pyrus L. total lipids are determined by mountain ecosystem conditions. Plant Physiol. Biochem..

[32] Budge S.M., Barry C. (2019). Determination of squalene in edible oils by transmethylation and GC analysis. MethodsX.

[33] Lyons J.M., Wheaton T.A., Pratt H.K. (1964). Relationship between the Physical Nature of Mitochondrial Membranes and Chilling Sensitivity in Plants. Plant Physiol..

[34] Janson J., Reinders M.C., Valkering A.G.M., Van Tuyl J.M., Keijzer C.J. (1994). Pistil Exudate Production and Pollen Tube Growth in *Lilium longiflorum* Thunb.. Ann. Bot..

[35] Reszczyńska E., Hanaka A. (2020). Lipids Composition in Plant Membranes. Cell Biochem. Biophys..

[36] Zhukov A. (2021). V On Qualitative Composition of Membrane Lipids in Plant Cells. Russ. J. Plant Physiol..

[37] Breygina M., Voronkov A., Ivanova T., Babushkina K. (2023). Fatty acid composition of dry and germinating pollen of Gymnosperm and Angiosperm plants. Int. J. Mol. Sci..

[38] Wolters-Arts M., Van Der Weerd L., Van Aelst A.C., Van Der Weerd J., Van As H., Mariani C. (2002). Water-conducting properties of lipids during pollen hydration. Plant. Cell Environ..

[39] Okolo E.C., Abigor R.D. (2006). Components Of The Stigmatic Exudate Of Raphia hookeri, Mann And Wendl. J. Agric. For. Soc. Sci..

[40] Kandasamy M.K., Vivekanandan M. (1983). Biochemical composition of stigmatic exudate of *Eichhorniacrassipes* (Mart.) solms. Aquat. Bot..

[41] Vogel S. Flowers offering fatty oil instead of nectar. Proceedings of the Abstracts XIth International Botany Congress.

[42] Baker H.G., Baker I., Opler P.A., Brantjes N.B.M. (1974). Stigmatic exudates and pollination. Pollination and Dispersal.

[43] Lord E.M., Webster B.D. (1979). The stigmatic exudate of *Phaseolus vulgaris* L.. Bot. Gaz..

[44] Stevenson P.C., Nicolson S.W., Wright G.A. (2017). Plant secondary metabolites in nectar: Impacts on pollinators and ecological functions. Funct. Ecol..

[45] Goldman M.H., Goldberg R.B., Mariani C. (1994). Female sterile tobacco plants are produced by stigma-specific cell ablation. EMBO J..

[46] Lush W.M., Grieser F., Wolters-Arts M. (1998). Directional guidance of *Nicotiana alata* pollen tubes in vitro and on the stigma. Plant Physiol..

[47] Lipp J. (1990). Detection and origin of abscisic acid and proline in honey. Apidologie.

[48] Davis A.R., Gunning B.E.S. (1993). The modified stomata of the floral nectary of *Viciafaba* L. 3. Physiological aspects, including comparisons with foliar stomata. Bot. Acta.

[49] Kovaleva L.V., Zakharova E.V., Minkina Y.V., Vvoronkov A.S. (2009). Effects of flavonols and phytohormones on germination and growth of petunia male gametophyte. Allelopath. J..

[50] Guan Y., Guo J., Li H., Yang Z. (2013). Signaling in pollen tube growth: Crosstalk, feedback, and missing links. Mol. Plant.

[51] Breygina M., Voronkov A., Galin I., Akhiyarova G., Polevova S., Klimenko E., Ivanov I., Kudoyarova G. (2022). Dynamics of endogenous levels and subcellular localization of ABA and cytokinins during pollen germination in spruce and tobacco. Protoplasma.

[52] Kovaleva L., Voronkov A., Zakharova E., Minkina Y., Timofeeva G., Andreev I. (2016). Regulation of Petunia pollen tube growth by phytohormones: Identification of their potential targets. J. Agric. Sci. Technol. A.

[53] Shi P., Hua W., Htwe Y.M., Zhang D., Li J., Wang Y. (2021). Abscisic Acid Improves Linoleic Acid Accumulation Possibly by Promoting Expression of EgFAD2 and Other Fatty Acid Biosynthesis Genes in Oil Palm Mesocarp. Front. Plant Sci..

[54] Huo K., Shui L., Mai Y., Zhou N., Liu Y., Zhang C., Niu J. (2020). Effects of exogenous abscisic acid on oil content, fatty acid composition, biodiesel properties and lipid components in developing Siberian apricot (*Prunus sibirica*) seeds. Plant Physiol. Biochem..

[55] Melo-Espinosa E.A., Sánchez-Borroto Y., Errasti M., Piloto-Rodríguez R., Sierens R., Roger-Riba J., Christopher-Hansen A. (2014). Surface Tension Prediction of Vegetable Oils Using Artificial Neural Networks and Multiple Linear Regression. Energy Procedia.

[56] Corbet S.A., Willmer P.G., Beament J.W.L., Unwin D.M., Prŷs-Jones O.E. (1979). Post-secretory determinants of sugar concentration in nectar. Plant. Cell Environ..

[57] Post-Beittenmiller D. (1996). Biochemistry and molecular biology of wax production in plants. Annu. Rev. Plant Physiol. Plant Mol. Biol..

[58] Posé D., Castanedo I., Borsani O., Nieto B., Rosado A., Taconnat L., Ferrer A., Dolan L., Valpuesta V., Botella M.A. (2009). Identification of the Arabidopsis dry2/sqe1-5 mutant reveals a central role for sterols in drought tolerance and regulation of reactive oxygen species. Plant J..

[59] Balusamy S.R., Rahimi S., Yang D.-C. (2019). Characterization of squalene-induced PgCYP736B involved in salt tolerance by modulating key genes of abscisic acid biosynthesis. Int. J. Biol. Macromol..

[60] Cowan A.K., Railton I.D. (1987). The biosynthesis of abscisic acid in a cell-free system from embryos of *Hordeum vulgare*. J. Plant Physiol..

[61] Hata S., Sanmiya K., Kouchi H., Matsuoka M., Yamamoto N., Izui K. (1997). cDNA cloning of squalene synthase genes from mono-and dicotyledonous plants, and expression of the gene in rice. Plant Cell Physiol..

[62] Zhang M., Wang S., Yin J., Li C., Zhan Y., Xiao J., Liang T., Li X. (2016). Molecular cloning and promoter analysis of squalene synthase and squalene epoxidase genes from *Betula platyphylla*. Protoplasma.

[63] Wang Z., Guo H., Zhang Y., Lin L., Cui M., Long Y., Xing Z. (2019). DNA methylation of farnesyl pyrophosphate synthase, squalene synthase, and squalene epoxidase gene promoters and effect on the saponin content of *Eleutherococcus senticosus*. Forests.

[64] Yin J., Li X., Zhan Y., Li Y., Qu Z., Sun L., Wang S., Yang J., Xiao J. (2017). Cloning and expression of BpMYC4 and BpbHLH9 genes and the role of BpbHLH9 in triterpenoid synthesis in birch. BMC Plant Biol..

[65] Yang L., Yang L., Lan Y., Zhao Y., Han M., Yang L. (2020). Exogenous abscisic acid reduces saikosaponin accumulation by inhibiting saikosaponin synthesis pathway gene expression under drought stress in *Bupleurum chinense* DC. Ind. Crops Prod..

[66] Romero P., Lafuente M.T. (2021). The combination of abscisic acid (ABA) and water stress regulates the epicuticular wax metabolism and cuticle properties of detached citrus fruit. Int. J. Mol. Sci..

[67] Mansouri H., Asrar Z., Szopa J. (2009). Effects of ABA on primary terpenoids and Δ9-tetrahydrocannabinol in *Cannabis sativa* L. at flowering stage. Plant Growth Regul..

[68] Mansouri H., Asrar Z. (2012). Effects of abscisic acid on content and biosynthesis of terpenoids in *Cannabis sativa* at vegetative stage. Biol. Plant..

[69] Micera M., Botto A., Geddo F., Antoniotti S., Bertea C.M., Levi R., Gallo M.P., Querio G. (2020). Squalene: More than a Step toward Sterols. Antioxidants.

