# Supplementary material for: Plant Hormone and Fatty Acid Screening of Nicotiana tabacum and Lilium longiflorum Stigma Exudates
Source: Biomolecules. 2023 Aug 27;13(9):1313. doi: 10.3390/biom13091313 (PMC10526190; doi:10.3390/biom13091313)
Supplement: Supplementary file 1 [file biomolecules-13-01313-s001.zip › biomolecules-2529228-supplementary.pdf]

Supplementary material

Plant Hormone and Fatty Acid Screening of *Nicotiana tabacum* and *Lilium longiflorum* Stigma Exudates

Maria Breygina, Dmitry Kochkin, Alexander Voronkov, Tatiana Ivanova, Ksenia Babushkina and Ekaterina Klimenko

List of supplementary material:

Figure S1. A representative UPLC-ESI-MS chromatogram (total ion current, base peak ion (BPI) mode, positive ions; *method 1*) of a model mixture of standard samples of phytohormones and structurally similar metabolites.

Figures S2–S6. Screening for indole-3-acetic acid (S2), indole-3-butyric acid (S3), 6-benzylaminopurine (S4), trans-zeatin (S5),and jasmonic acid (S6), in tobacco stigma exudate (stage 3).;

Figure S7. Screening for the presence of major phytohormones in tobacco stigma exudate (stage 3, sample collected in the summer of 2022).

Figure S8. ABA in the stigma exudate of tobacco (stage 2).

Figure S9. Screening for ABA in tobacco stigma exudate (*method 2*).

Figure S10. Mass spectra of the chromatographic peak of the standard sample of ABA (lower panel) and the chromatographic peak with a retention time of 3.49 min in the chromatogram of tobacco stigma exudate (upper panel).

Figure S11. Screening for the presence of major phytohormones in lily stigma exudate (stage 3).

Figure S12. Squalene effect on tobacco pollen germination in vitro.

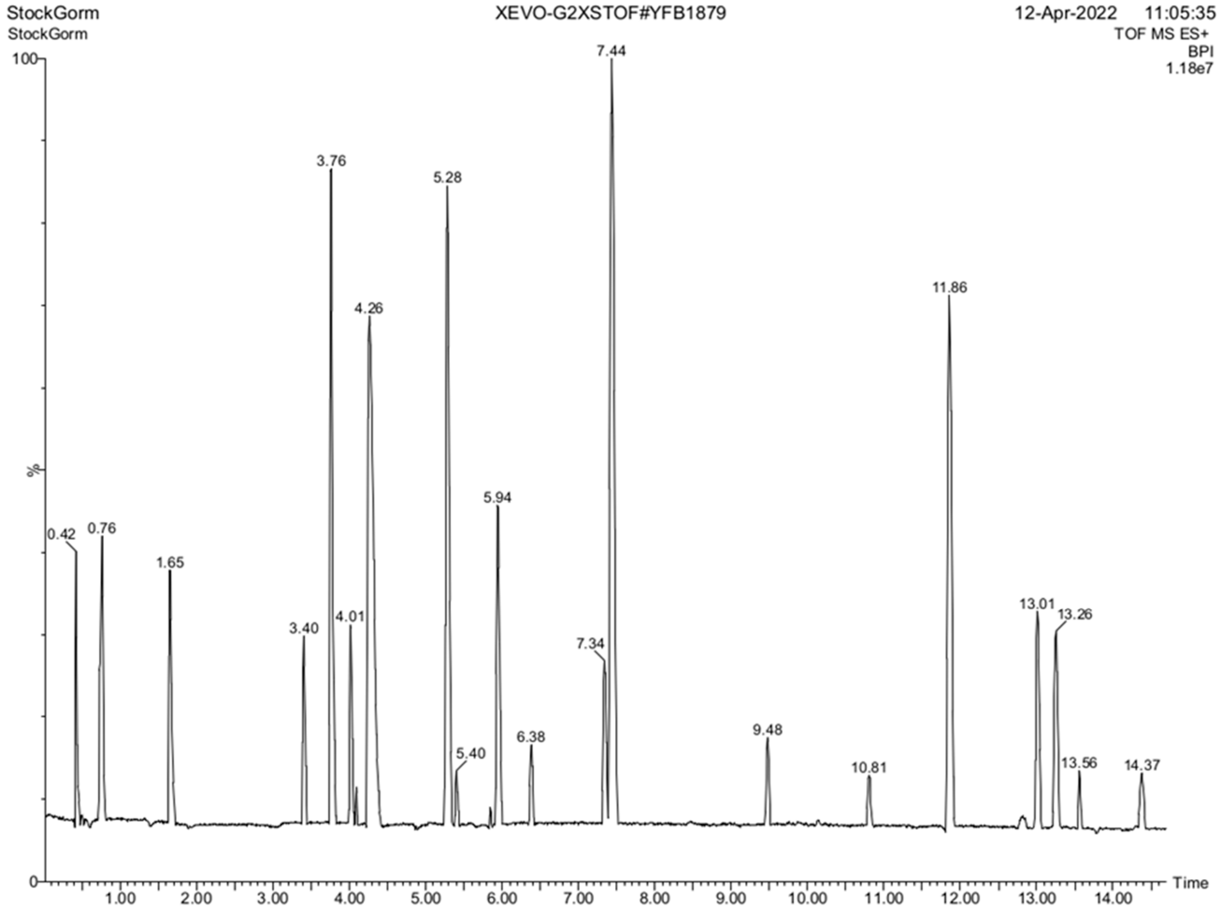

Figure S1. A representative UPLC-ESI-MS chromatogram (total ion current, base peak ion (BPI) mode, positive ions; *method 1*) of a model mixture of standard samples of phytohormones and structurally similar metabolites. Identification of chromatographic peaks by retention time: spermine (retention time—0.42 min), adenine (0.76 min), hordenine (1.65 min), salicylic acid (3.40 min), trans-zeatin (3.76 min), kinetin (5.28 min), 2,4-dichlorophenoxyacetic acid (5.94 min),picloram (6.38 min), 2-phenylethyl-glucoside(7.34 min),6-benzylaminopurine (7.44 min), indole-3-acetic acid (9.48 min), salicin (10.81 min), ABA (11.86 min), indole-3-butyric acid (13.01 min), jasmonic acid (13.26 min), 5-methyltryptophan (13.56 min), and indigo (14.37 min). X—t, min; Y– detector signal, relative intensity (RI), %.

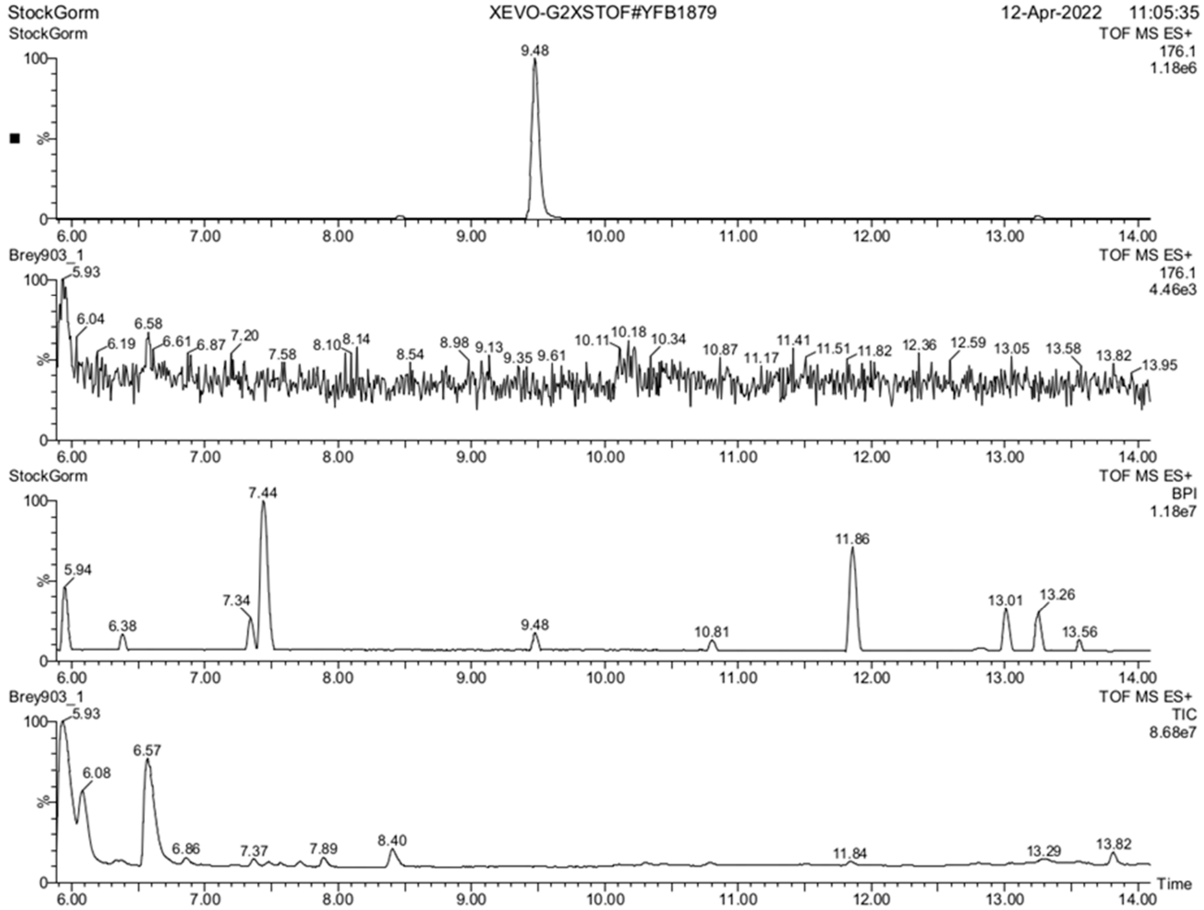

Figure S2. Screening for indole-3-acetic acid in tobacco stigma exudate (stage 3). UPLC-ESI-MS chromatograms (total ion current (TIC) and BPI modes, positive ions; *method 1*) of tobacco stigma exudate (lower panel) and a model mixture of standard phytohormone samples (second panel from the bottom), as well as the results of filtering these signals to the m/z value of the $[M + H]^+$ ion of the hormone (the third and fourth panels from the bottom for the exudate and the model mixture of standards, respectively). X—t, min; Y—detector signal, RI, %;

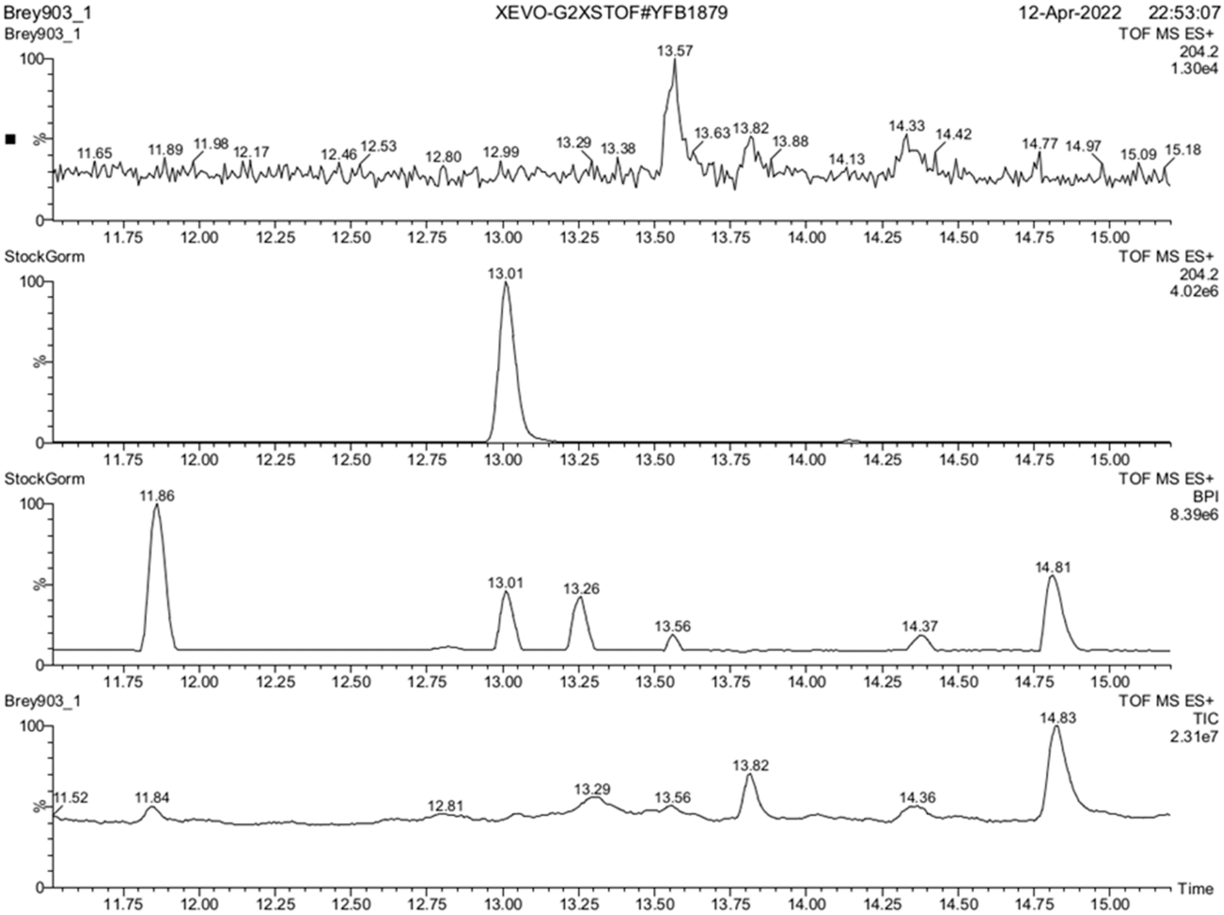

Figure S3. Screening for indole-3-butyric acid in tobacco stigma exudate (stage 3). UPLC-ESI-MS chromatograms (total ion current (TIC) and BPI modes, positive ions; *method 1*) of tobacco stigma exudate (lower panel) and a model mixture of standard phytohormone samples (second panel from the bottom), as well as the results of filtering these signals to the m/z value of the $[M + H]^+$ ion of the hormone (the third and fourth panels from the bottom for the exudate and the model mixture of standards, respectively). X—t, min; Y—detector signal, RI, %;

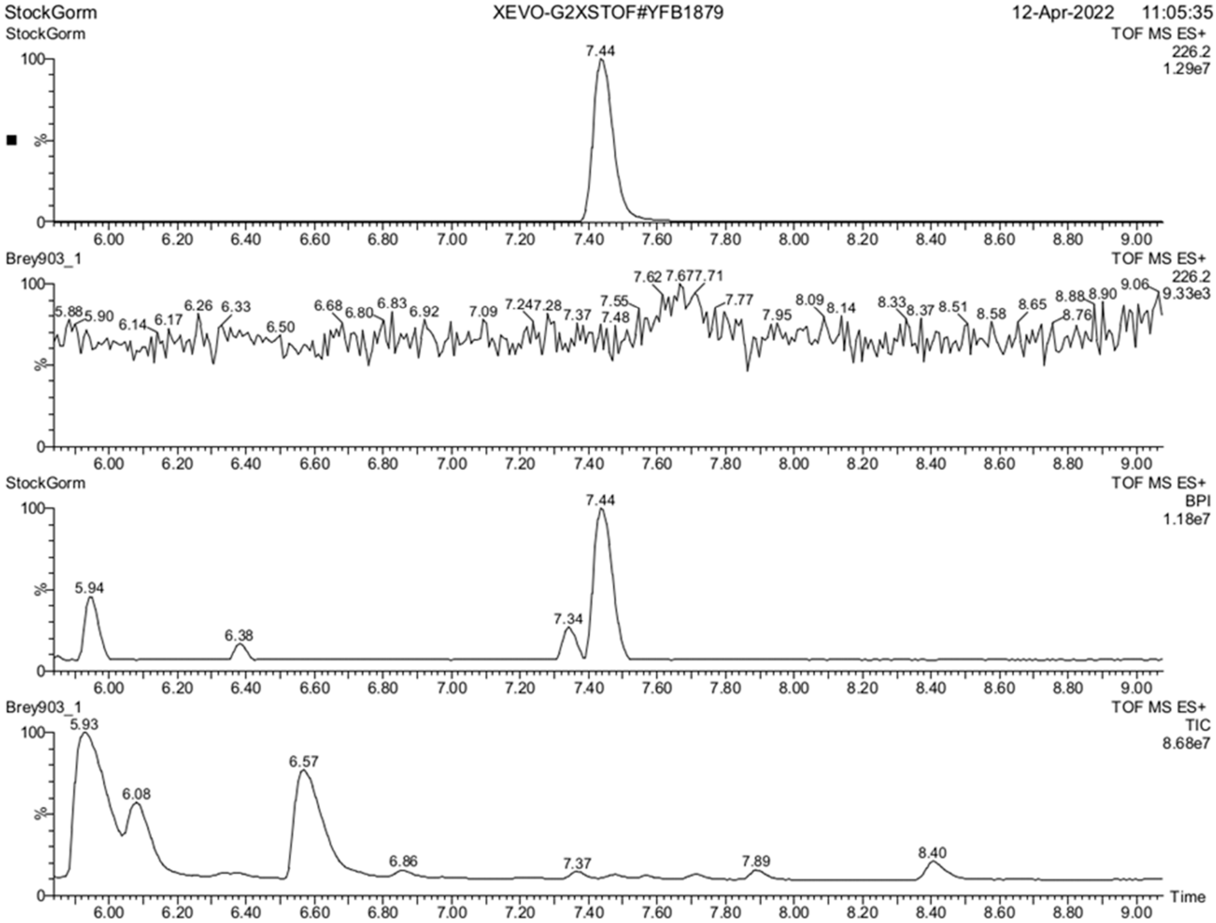

Figure S4. Screening for 6-benzylaminopurine in tobacco stigma exudate (stage 3). UPLC-ESI-MS chromatograms (total ion current (TIC) and BPI modes, positive ions; *method 1*) of tobacco stigma exudate (lower panel) and a model mixture of standard phytohormone samples (second panel from the bottom), as well as the results of filtering these signals to the m/z value of the $[M + H]^+$ ion of the hormone (the third and fourth panels from the bottom for the exudate and the model mixture of standards, respectively). X—t, min; Y—detector signal, RI, %;

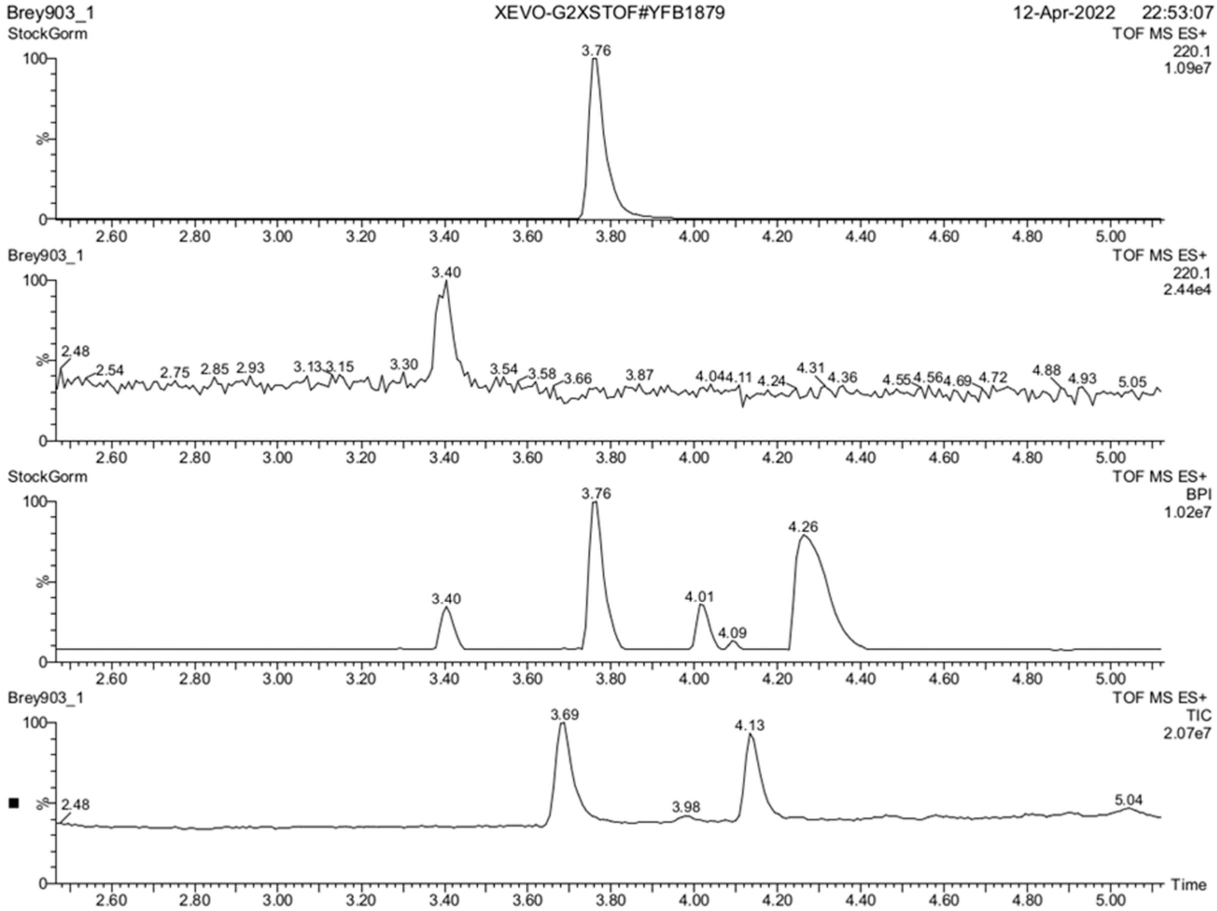

Figure S5. Screening for trans-zeatin in tobacco stigma exudate (stage 3). UPLC-ESI-MS chromatograms (total ion current (TIC) and BPI modes, positive ions; *method 1*) of tobacco stigma exudate (lower panel) and a model mixture of standard phytohormone samples (second panel from the bottom), as well as the results of filtering these signals to the m/z value of the $[M + H]^+$ ion of the hormone (the third and fourth panels from the bottom for the exudate and the model mixture of standards, respectively). X—t, min; Y—detector signal, RI, %;

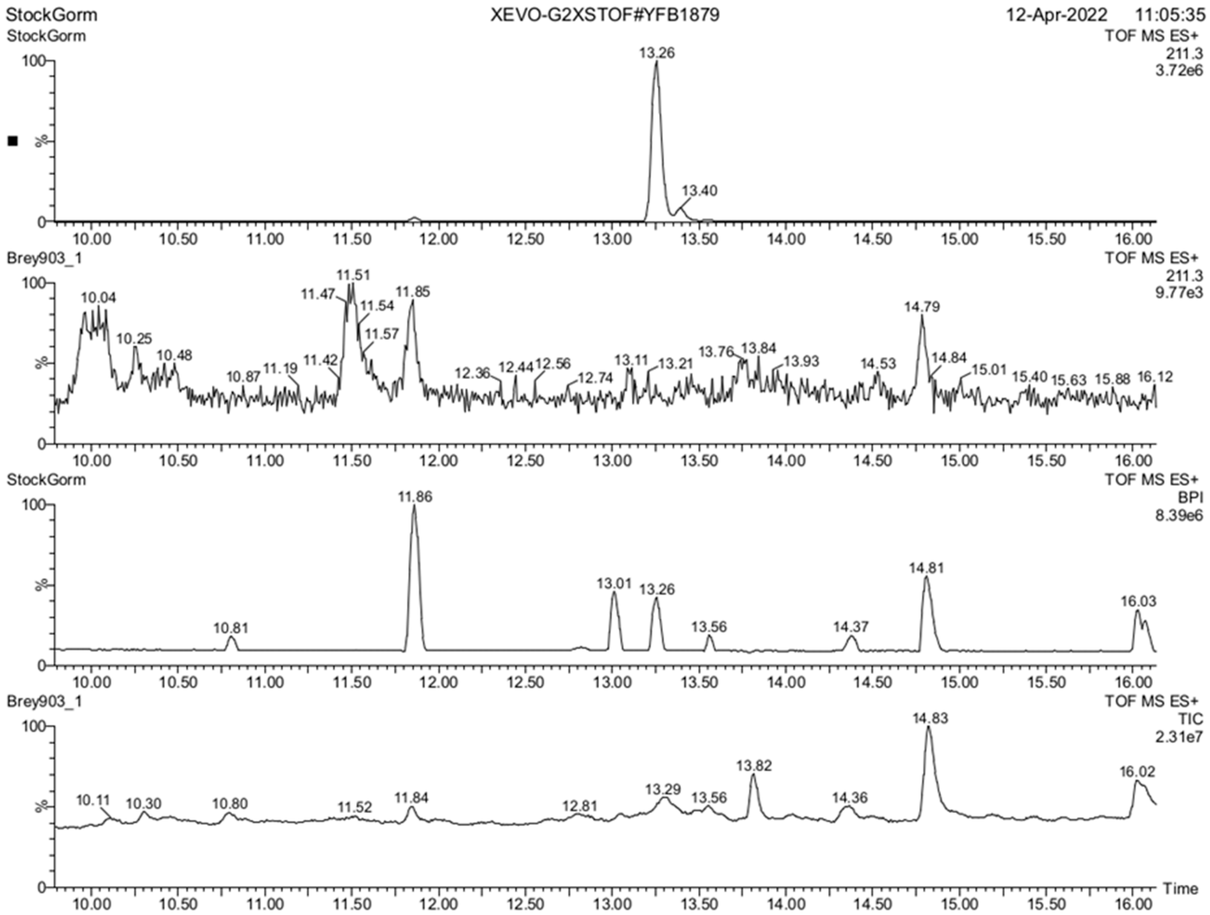

Figure S6. Screening for jasmonic acid in tobacco stigma exudate (stage 3). UPLC-ESI-MS chromatograms (total ion current (TIC) and BPI modes, positive ions; *method 1*) of tobacco stigma exudate (lower panel) and a model mixture of standard phytohormone samples (second panel from the bottom), as well as the results of filtering these signals to the m/z value of the $[M + H]^+$ ion of the hormone (the third and fourth panels from the bottom for the exudate and the model mixture of standards, respectively). X—t, min; Y—detector signal, RI, %;

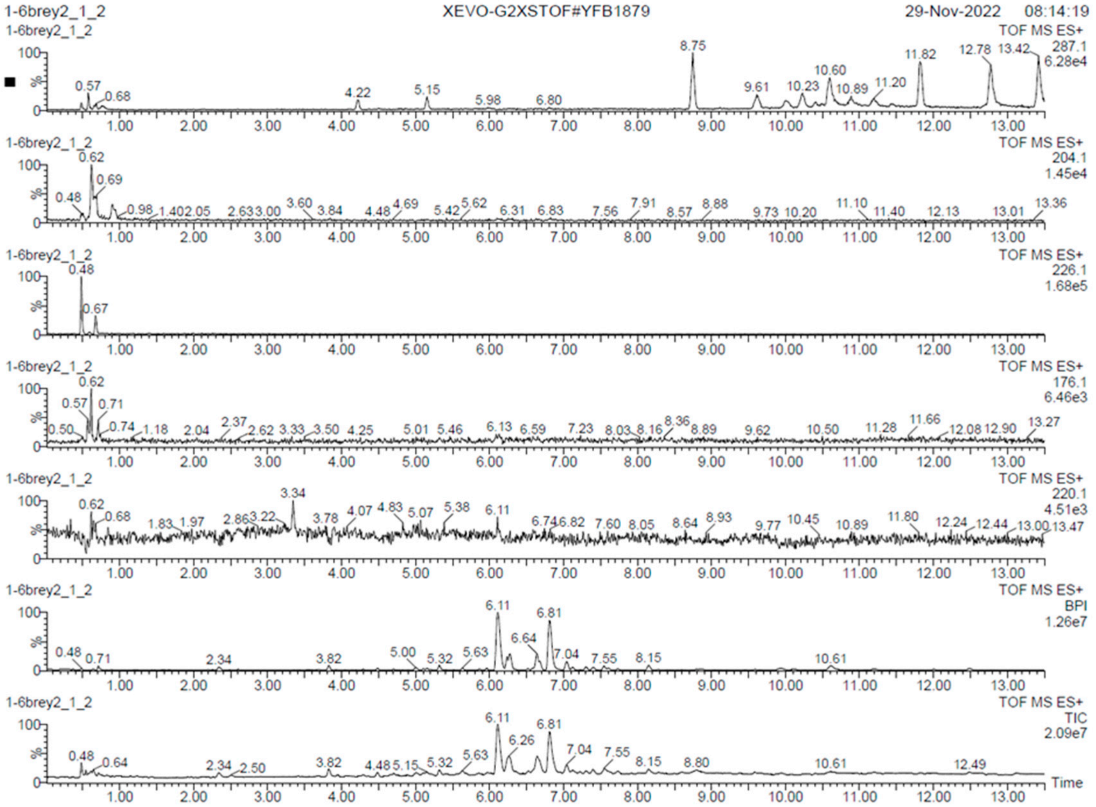

Figure S7. Screening for the presence of major phytohormones in tobacco stigma exudate (stage 3, sample collected in the summer of 2022). UPLC-ESI-MS chromatogram (total ion current (TIC and BPI modes), positive ions; *method 1*) of exudate (first and second panel from the bottom), results of filtering the TIC signal by m/z value for the following ions: m/ z 220.1—[M + H]$^+$ trans-zeatin ion (third panel from the bottom); m/z 176.1—[M + H]$^+$ion of indole-3-acetic acid (panel fourth from bottom); m/z 226.2—ion [M + H]$^+$ 6-benzylaminopurine (fifth panel from the bottom); m/z 204.2—[M + H]$^+$ ion of indole-3-butyric acid (sixth panel from the bottom); m/z 287.1—[M + Na]$^+$ ion of ABA (panel seventh from the bottom). X—t, min; Y—detector signal, RI, %. The chromatographic peak of ABA has a retention time of 11.82 min;

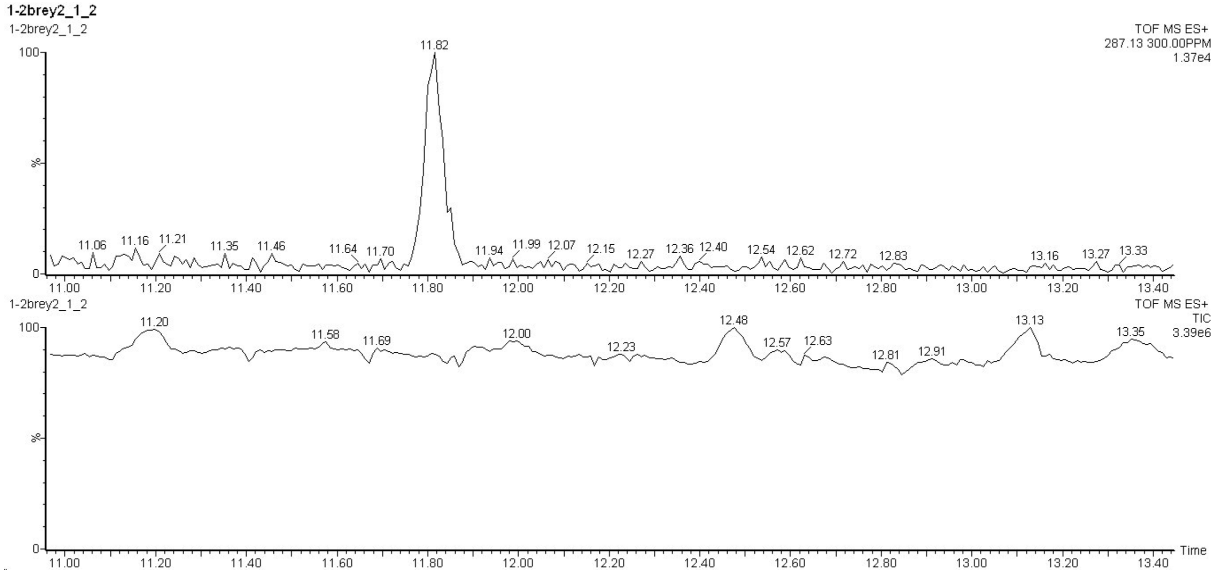

Figure S8. ABA in the stigma exudate of tobacco (stage 2). UPLC-ESI-MS chromatogram (total ion current, positive-ion mode) obtained by *Method 1* of tobacco stigma exudate. Upper panel—results of signal filtering by m/z value (value 287.1) of [M + Na]$^+$ ion of ABA; Lower panel—primary signal. X—t, min; Y—detector signal, relative intensity (RI), %;

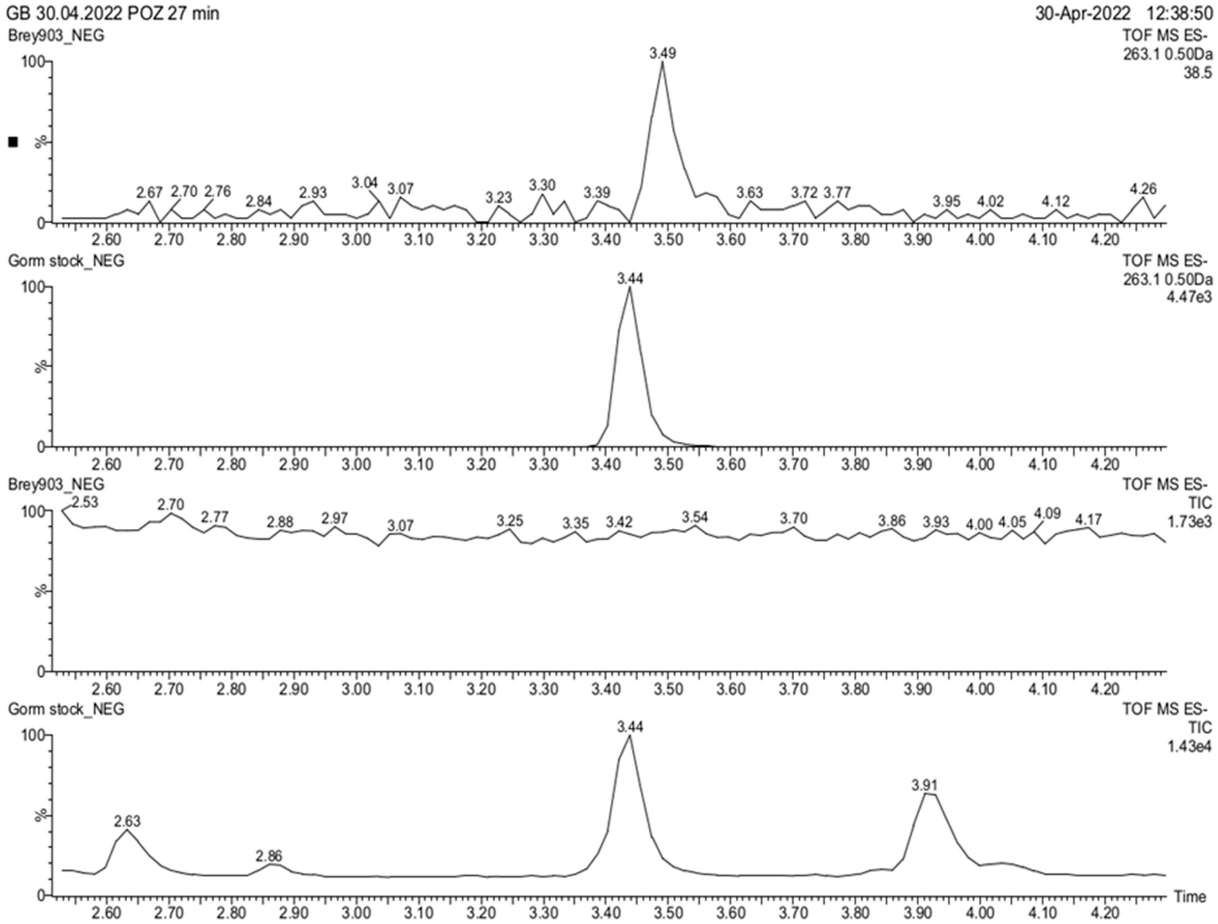

Figure S9. Screening for ABA in tobacco stigma exudate (*method 2*). The UPLC-ESI-MS chromatograms (total ion current, negative ions) of tobacco stigma exudate (second panel from the bottom) and the model mixture of standard phytohormone samples (bottom panel) are presented, as well as the results of filtering these signals by m/z value (value 263.1) $[M-H]^-$ABA ion (third and fourth panels from the bottom for the model mixture of standards and exudate, respectively). X—t, min; Y—detector signal, RI, %;

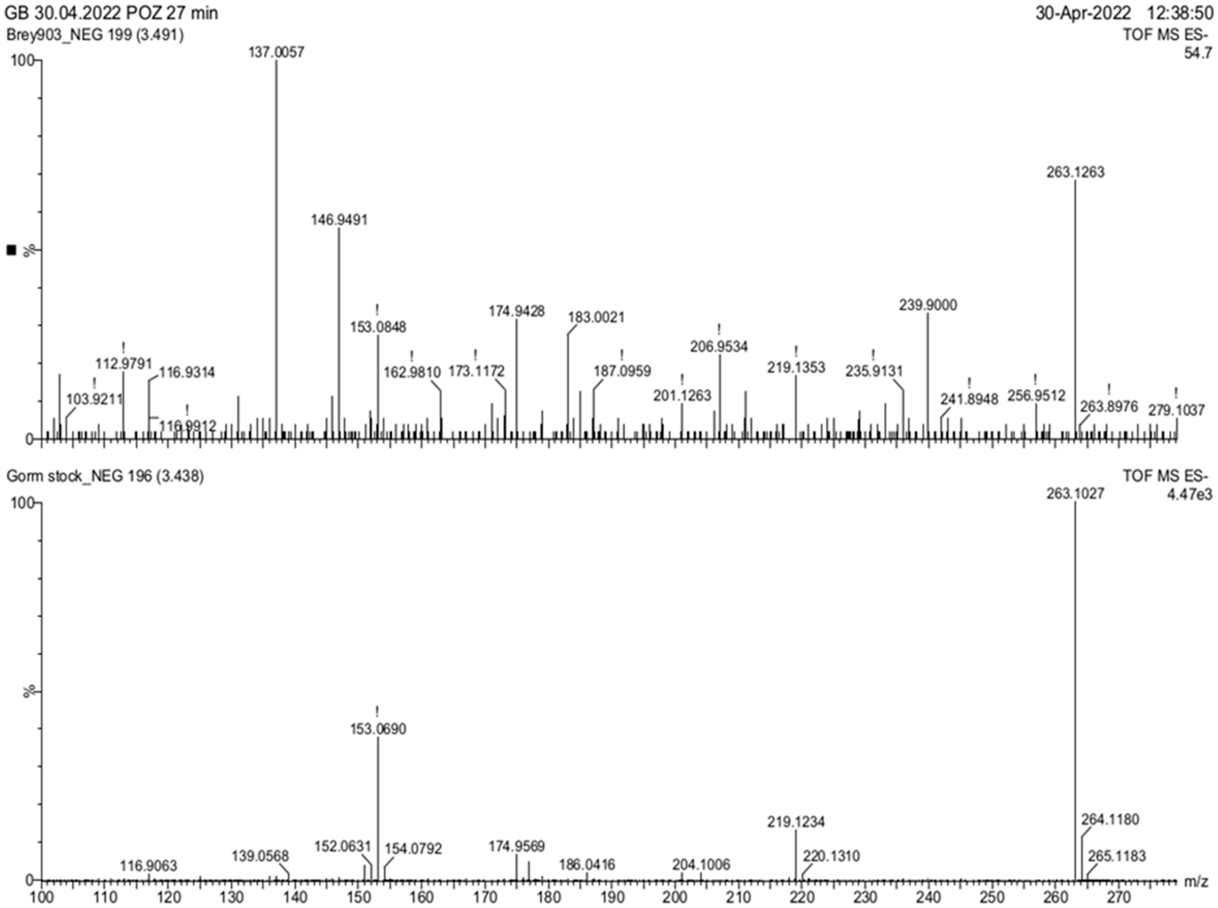

Figure S10. Mass spectra of the chromatographic peak of the standard sample of ABA (lower panel) and the chromatographic peak with a retention time of 3.49 min in the chromatogram of tobacco stigma exudate (upper panel). *Method 2*. X—m/z; Y—detector signal, RI, %;

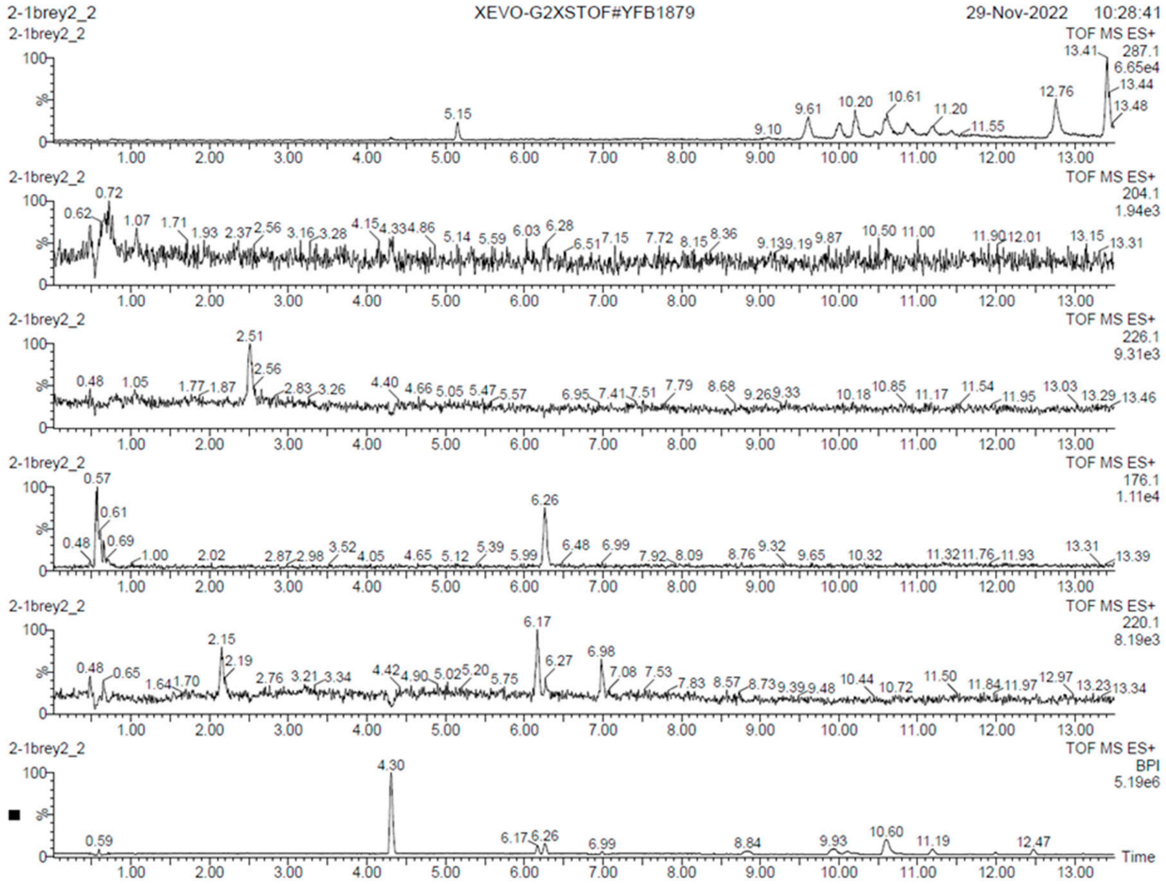

Figure S11. Screening for the presence of major phytohormones in lily stigma exudate (stage 3). UPLC-ESI-MS chromatogram (TIC mode, positive ions; *method 1*) of the exudate (first panel from the bottom), results of filtering the TIC signal by m/z value for the following ions: m/z 220.1—ion $[M + H]^+$ trans-zeatin (panel second from bottom); m/z 176.1—$[M + H]^+$ ion of indole-3-acetic acid (third panel from the bottom); m/z 226.2—ion $[M + H]^+$ 6-benzylaminopurine (fourth panel from the bottom); m/z 204.2—$[M + H]^+$ ion of indole-3-butyric acid (panel fifth from the bottom); and m/z 287.1—$[M + Na]^+$ ion of ABA (sixth panel from the bottom). X—t, min; Y—detector signal, RI, %;

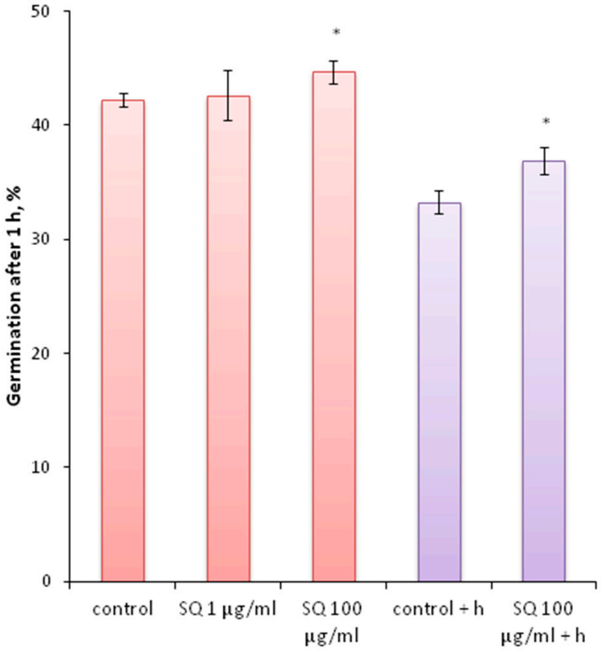

Figure S12. Squalene effect on tobacco pollen germination in vitro. Squalene added to pollen suspensions germinating in vitro in concentration close to the one found in stigma exudate (1 µg/mL had no effect on germination efficiency, and 100 µg/mL had a weak stimulating effect both with pure squalene in water and squalene diluted in hexane). *—$p < 0.05$ (Mann–Whitney test).
